# Effects of Tumor Localization, Age, and Stage on the Outcomes of Gastric and Colorectal Signet Ring Cell Adenocarcinomas

**DOI:** 10.3390/cancers15030714

**Published:** 2023-01-24

**Authors:** Matthew G. K. Benesch, Alexander Mathieson, Shalana B. L. O’Brien

**Affiliations:** 1 Department of Surgical Oncology, Roswell Park Comprehensive Cancer Center, Buffalo, NY 14263, USA; 2 Discipline of Surgery, Faculty of Medicine, Memorial University of Newfoundland, St. John’s, NL A1B 3V6, Canada

**Keywords:** cancer, chemotherapy, epidemiology, histopathology, radiotherapy, surgery, survival outcomes

## Abstract

**Simple Summary:**

Signet ring cell adenocarcinomas are an extremely rare type of cancer that arise primarily in the stomach and colon. These cancers tend to present at an earlier age with more advanced disease. It is, however, not known if disease progression depends on the location within the stomach or the colon from where it arises. Across both sexes and all ages, these cancers occur more often in the distal rather than the proximal stomach, compared to regular gastric cancers. For early disease stages, younger patients have similar outcomes to regular gastric cancers, but there are worse outcomes in older patients. In colorectal cancer patients with signet ring cells, outcomes are worse universally across all ages and stages, and most of these cancers arise in the right colon. Mortality, however, is worse in cancers arising from the left colon and rectum. This work offers insights into factors that better characterize their pathology.

**Abstract:**

Signet ring cell adenocarcinomas (SRCCs) are a rare histological adenocarcinoma subtype, classically thought to have a worse prognosis than conventional adenocarcinomas. The majority of these cancers occur in the stomach, colon, and rectum. Their rarity means that most epidemiological studies into their pathology are often underpowered, and interpretations from these reports are mixed. In this study, we use the Surveillance, Epidemiology, and End Results Program (SEER) database to examine the effects of tumor localization, age, and stage on gastric and colorectal cancer outcomes. For early onset localized and regional gastric cancers, SRCCs have the same overall risk of mortality compared to conventional adenocarcinomas. Over the age of 50 years, SRCCs have worse outcomes across all stages. Gastric SRCCs are 2–3-fold more likely in younger patients, and more heavily favor the distal stomach. Like conventional adenocarcinomas, proximal gastric SRCCs have decreased survival. Across all ages, stages, and locations, colorectal SRCCs have worse outcomes. SRCCs favor the right colon, but outcomes are significantly worse for the left colon and rectal cancers. Relative to adenocarcinomas, colorectal SRCCs have the worst outcomes in younger patients. Overall, these results provide insights into SRCC disease patterns that cannot be surmised outside of population-level data.

## 1. Introduction

Signet ring cell adenocarcinomas (SRCCs) are a rare histological subtype typically associated with earlier age and a higher stage of disease at diagnosis, resulting in an overall poorer prognosis compared to conventional adenocarcinomas. A signet ring cell is a cancer cell with excessive intracytoplasmic mucin, resulting in the cell nucleus being eccentrically compressed against the cytoskeleton [1]. A cancer is defined as an SRCC if greater than 50% of cancer cells within the tumor contain this signet ring cell morphology [2].

Despite being described in the medical literature for over 70 years and formally codified as a histologically distinct cancer subtype since 1990, the rarity of this cancer diagnosis has largely excluded the subtype from meaningful prospective clinical studies [3]. SRCCs comprise less than 0.5% of all solid tumor diagnoses, and hence most published work on them comes either from small series or retrospective analyses of large or national cancer databases [3]. In fact, SRCCs are often grouped with other rare cancer subtypes as exclusion criteria in large cohort studies [4]. We have previously studied the epidemiology of SRCCs, and showed that their distribution heavily favors gastric and colorectal sites [3]. Nearly 60% of all SRCCs are gastric, with colorectal comprising the second largest group at about 20% [3]. Nearly 17% of all gastric cancers are SRCCs, while only 1% of colorectal cancers are SRCCs [3]. This contrasts with conventional adenocarcinomas, which comprise approximately 60% of all carcinomas of both sites [3].

Due in part to the rarity of this disease, most studies on SRCCs are comprised of small cohorts that are both single-institutional analyses and retrospective in nature. Attempts have been made to examine the effects of age, tumor localization, and stage at presentation on prognosis. For example, in a review of over 34,000 consecutive cases of colorectal cancer (CRC) in a large tertiary center, 17.9% of early onset (age < 50 years) CRCs were poorly differentiated with mucinous or signet ring cell features compared to 11.6% in the late onset (age > 50 years) group [5]. A recent meta-analysis of 30 articles and over 1 million CRC patients concluded that SRCCs occur at a younger age with a higher rate of right-sided lesions, and more advanced stages at presentation with high reoccurrence rates [6]. Gastric SRCC research, however, tends to be less concordant. Some studies have suggested that in early gastric cancer, SRCC patients have a more favorable prognosis than the intestinal type, but this trend reverses in higher stage disease [7,8]. Higher rates of SRCCs tend to occur in females under 45 years old, resulting is a worse overall survival compared to other histologies and the male sex [9]. Nevertheless, there are multiple reports, particularly from Asian centers, demonstrating that pre-menopausal women may have a survival advantage over post-menopausal women and men [10,11]. However, these studies tend to label gastric cancers as SRCCs when there is any signet ring cell component. When outcomes are dichotomized between those with <50% SRCCs and >50% SRCCs, the apparent survival benefit largely disappears in patients with higher signet ring cell components [10]. Finally, gastric cancers involving the upper third of the stomach tend to have poorer prognosis, regardless of the stage at presentation [12], but SRCCs tend to have higher presentation rates in the middle and lower stomach [13,14]. The relationship between SRCC pathology and gastric location is essentially unknown.

In this study, we use the Surveillance, Epidemiology, and End Results (SEER) database to provide a standardized characterization of the outcomes of the most common SRCCs, gastric and colorectal, by anatomical location, age (early onset versus late onset), sex, and stage at presentation. These findings are then compared to nonvariant adenocarcinomas. The SEER database is a population-based cancer registry run by the National Cancer Institute, and it captures nearly all cancer cases through regional registries for over one-third of the United States population, some of which have existed for over forty years [15]. Population-level data is required to perform a meaningful subgroup analysis that is limited by the extremely low incidence of SRCCs.

## 2. Materials and Methods

### 2.1. Patient Selection

The National Cancer Institute’s SEER database comprised from 18 SEER cancer registries was employed using data from 1992–2016, as previously described [3,16]. Data release from the SEER database does not require informed patient consent or review by an institutional review board. The SEER database was accessed and searched in compliance with signed user agreements. Exclusion criteria and all variables are defined previously [3,16].

### 2.2. Statistical Analysis

All selected data from SEER cancer registries were imported into Stata 15.1 (StataCorp LLC, College Station, TX, USA) for statistical analysis. A complete case analysis was completed after the variable definition described previously [3,16]. We then excluded cases for which tumors were not localizable. The resulting ICD-O-3 codes used for patient selection are detailed in Table S1.

Baseline patient characteristics were compared with the *t* and *χ*^2^ tests for continuous and categorical variables, respectively. Univariate and multivariable Cox proportional hazard regressions were used to determine the association of mortality with cancer histology type, adjusting for age, sex, race, detection stage, grade differentiation, surgery, radiotherapy, and chemotherapy. All hazard ratios are calculated with 95% confidence intervals. The use of surgery, radiotherapy, and chemotherapy as treatment variables are binary. All *p*-values are two-sided, with a threshold of 0.05 to determine statistical significance. Survival curves were plotted using the Kaplan-Meier method, with *p*-values for survival curves generated by the log rank test. Graphs are plotted using Origin Pro 2022 (OriginLab Corporation, Northampton, MA, USA). Using SEER 18 (2000–2018) data with SEER*Stat 8.4.1 (Surveillance Research Program, National Cancer Institute, Calverton, MD, USA), incidence rates are calculated and age-adjusted to the 2000 United States standard population with the age variable recode < 1-year-old. Cause-specific survival and relative survival are both age standardized to the International Cancer Survival Standard 1-Age 15+ variable via the actuarial method, and Ederer II cumulative expected method for relative survival.

## 3. Results

### 3.1. Frequency, Adjusted Mortality, and Survival Trends for Gastric SRCCs

For localizable tumors, 66.7% of conventional gastric adenocarcinomas arise in the proximal stomach (cardia, fundus, and body), compared to 55.0% for SRCCs (Table 1). Within the proximal stomach, SRCCs are enriched in the body at 20.1% versus 11.8% for conventional adenocarcinomas. The majority of gastric SRCCs arise in the antrum at 39.4%, while the majority of gastric conventional adenocarcinomas are found in the cardia at 49.3% (Table 1).

The frequency distribution analysis is then repeated for the proximal and distal stomach localization dichotomized by sex and age grouping (<50 years old, ≥50 years old) (Table 2). In total, 9.2% and 7.7% of all gastric adenocarcinomas arise in the male and female patients under the age 50. When compared to SRCCs, the percentage more than doubles for males at 18.8% and triples for females at 22%. Among adenocarcinomas, when dichotomized by sex, nearly 75% of cases in male patients arise in the proximal stomach, as opposed to ~54% in females (Table 2). Particularly in males under 50 years old, for SRCCs, there is a substantial decrease in the proximal stomach percentage from 76.1% to 54.7% when compared to adenocarcinomas. For both the adenocarcinomas and SRCCs groupings, distant disease is the most common presentation in patients under 50 years old across all locations. In the over 50-year-old groups, rates of distant disease presentation are higher for SRCCs across all locations. Finally, with respect to incidence rates, the rate of adenocarcinomas is about 30-fold higher when comparing the older group to the younger group for both sexes (Table 2). The ratio decreases to about 10-fold for males with SRCCs and about 8-fold for females.

On both univariate and multivariable analyses, gastric SRCCs across all sites have worse overall survival compared to gastric adenocarcinomas (hazard ratios 1.14 and 1.11, respectively) (Table 3). Hazard ratios for tumors in both the proximal and distal stomach are not significantly different from these results. On further breakdown by anatomical location, the statistically significant hazard ratio is explained by tumors in both the cardia and antrum (1.20 and 1.09, respectively). There is no significant difference in tumors arising from the fundus, body, or pylorus. When these analyses are repeated on dichotomized age groups, across all sites, SRCC does not have a statistically significant effect on mortality on either univariable or multivariable adjustment (Table 3). Significance is again achieved in the over 50-year-old group and reflects the findings in the all ages column.

Tables S2 and S3 compare hazard ratios for individual sites relative to the cardia within adenocarcinoma and SRCCs groups. General trends are that diseases in the cardia and fundus tend to have worse mortality for both cancer histologies compared to other sites in the stomach.

**Figure 1:**
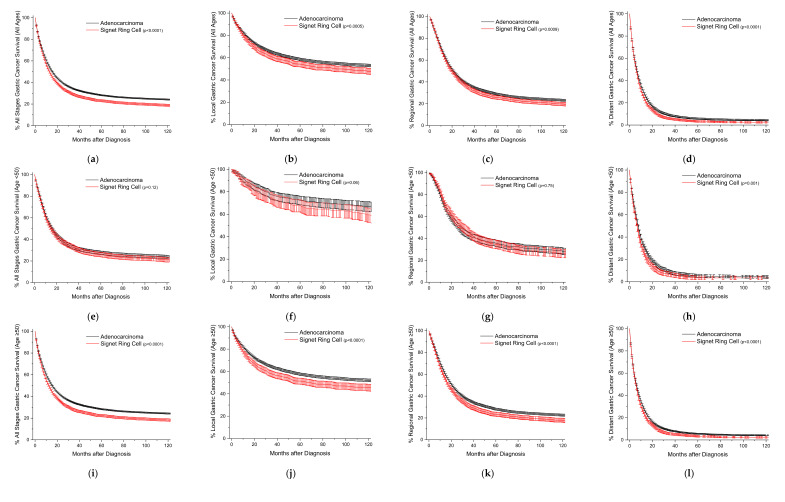
Kaplan-Meier survival curves for gastric cancer. All survivor functions are shown with 95% confidence intervals. (**a**) All ages, all stages. (**b**) All ages, local disease. (**c**) All ages, regional disease. (**d**) All ages, distant disease. (**e**) Age < 50, all stages. (**f**) Age < 50, local disease. (**g**) Age < 50, regional disease. (**h**) Age < 50, distant disease. (**i**) Age ≥ 50, all stages. (**j**) Age ≥ 50, local disease. (**k**) Age ≥ 50, regional disease. (**l**) Age ≥ 50, distant disease. *p*-values between curves by log-rank test.

Kaplan-Meier survival curves are plotted for both the age group and site of detection (Figure 1). When analyzed by all ages, SRCCs patients have worse survival, regardless of the stage of detection (Figure 1a–d). In the under 50-year-old group, there are no statistically significant differences between SRCCs and adenocarcinomas, except for distant disease (Figure 1e–h). In the 50-year-old and older age group, SRCCs have worse outcomes across all stages of the disease (Figure 1i–l). The median months of cause-specific survival for localized disease for adenocarcinomas versus SRCCs are 96.8 versus 69.2 months (Table S4). The differences in median survival between the two histologies decrease to 20.9 and 20.1 months for regional disease and to essentially the same at 6 months for distal disease. On analysis by site, proximal tumors have worse overall outcomes compared to distal tumors for both adenocarcinomas and SRCCs (Table S4). The survival difference between adenocarcinomas and SRCCs is most pronounced for localized disease with a median survival of 69.9 versus 35.4 months for proximal stomach cancers, and the median survival is not reached in either histological group by 10 years for distal gastric tumors (Table S4). For reference, this analysis is repeated dichotomously for ages <50 and ≥50 (Table S4), and relative survivals are also included (Table S5).

### 3.2. Frequency, Adjusted Mortality, and Survival Trends for Colorectal SRCCs

For localizable tumors, 33.4% of conventional colorectal adenocarcinomas arise in the right colon, compared to 55.3% for SRCCs (Table 4). Within the right colon, SRCCs arise primarily in the cecum at 23.5% of all colorectal SRCCs, compared to 16% for adenocarcinomas. Cancer rates in the transverse colon are essentially the same at about 7%. Overall rates of SRCCs are about 10% less in each of the left colon (29.6% versus 17.1%) and rectal region (30.3% versus 20.6%) relative to adenocarcinomas.

In the repeat analysis subgroup by sex and age, about 21% of adenocarcinomas arise in the right colon in both males and females under 50 years old, and for SRCCs, this rate is 36.8% in males and 49.4% in females (Table 5). In the ≥50-year-old group, for the adenocarcinomas, there is an increase to 30.1% in males and 39.8% in females, but for SRCCs, the percentages increase even further to 52.6% in males and 65.1% in females. Across all age groups and sexes, SRCCs present predominantly with distant disease, and this trend is most pronounced in the female <50-year-old category, at 56.4% in the right colon and 60.6% in the left colon. These percentages are about 15% less in males of the same age grouping. Localized disease rates for adenocarcinomas range from about 23% to about 33%, but range significantly less from 8–15% for SRCCs. Incidental rates are about 20-fold higher for the older group to the younger group for adenocarcinomas, and this rate decreases to about 10–15-fold for the SRCCs (Table 5).

On both univariate and multivariable analyses, colorectal SRCCs across all sites have worse overall survival compared to colorectal adenocarcinomas (hazard ratios 2.39 and 1.55, respectively) (Table 6). The hazard ratios are essentially similar within the two age groups but is statistically higher in the age <50-year-old group (2.99 and 1.78, respectively). Tumors arising in the left colon and rectal area have worse hazard ratios than right and transverse colon tumors (~1.8–2.1 versus ~1.2–1.4) (Table 6).

Tables S6 and S7 compare hazard ratios for individual sites relative to the transverse colon by adenocarcinoma and SRCC groupings. Adenocarcinomas arising in the rectum of those ≥50-years-old have a multivariable hazard ratio of about 1.2 relative to all other sites (Table S6). This same trend also occurs within the SRCC group (hazard ratio 1.6).

**Figure 2:**
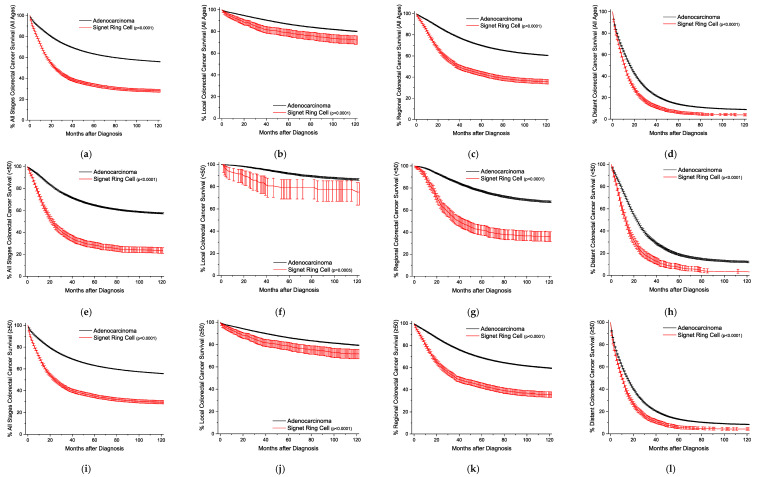
Kaplan-Meier survival curves for colorectal cancer. All survivor functions are shown with 95% confidence intervals. (**a**) All ages, all stages. (**b**) All ages, local disease. (**c**) All ages, regional disease. (**d**) All ages, distant disease. (**e**) Age < 50, all stages. (**f**) Age < 50, local disease. (**g**) Age < 50, regional disease. (**h**) Age < 50, distant disease. (**i**) Age ≥ 50, all stages. (**j**) Age ≥ 50, local disease. (**k**) Age ≥ 50, regional disease. (**l**) Age ≥ 50, distant disease. *p*-values between curves by log-rank test.

Kaplan-Meier survival curves are plotted for both age group and site of detection (Figure 2). SRCCs have significantly worse survival in every plot. When comparing the differences by stage, the largest spread between the curves occurs for regional disease (Figure 2). This trend holds across both age groups. For the localized disease, five-year cancer specific survival is about 85% for adenocarcinomas as opposed to about 77% for SRCCs (Table S8). For the regional disease, survival is about 70% versus 43% for the groups, respectively. In the distant disease, this survival decreases to about 14% and 5%, respectively. For reference, this analysis is repeated dichotomously for ages <50- and ≥50 years old (Table S8), and relative survivals are also included (Table S9).

**Figure 3:**
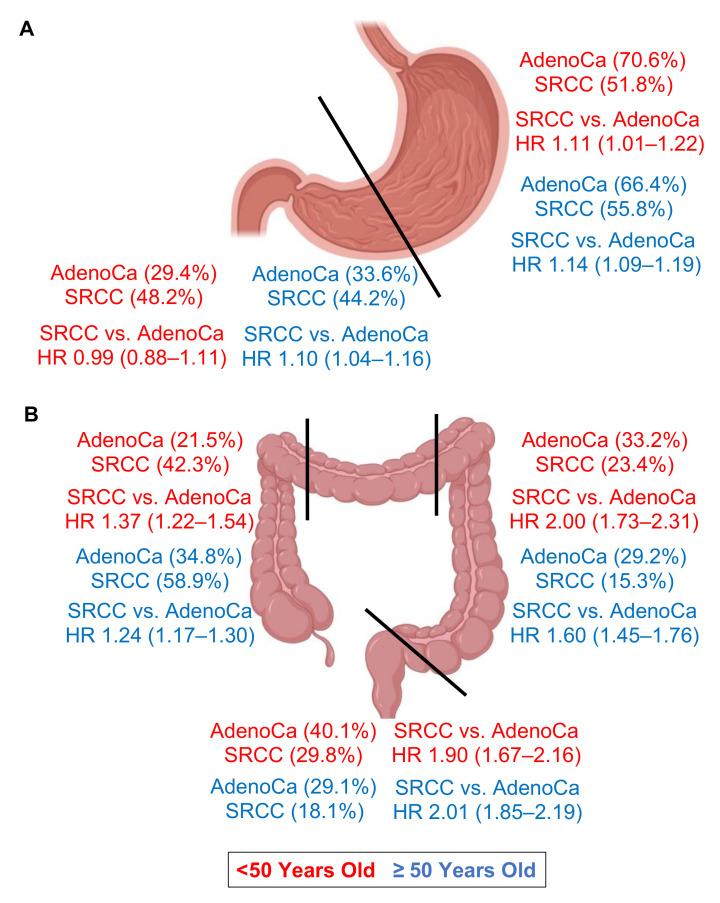
Graphical summary of key findings. (**A**) Percent distribution and hazard ratio comparing adenocarcinomas to signet ring cell adenocarcinomas in the proximal and distal stomach by age. (**B**) Percent distribution and hazard ratio comparing adenocarcinomas to signet ring cell adenocarcinomas in the right colon, left colon, and rectum by age. Red indicates values for patients < 50 years old, and blue for patients ≥ 50 years old. Hazard ratios display 95% confidence intervals. AdenoCa, adenocarcinoma; SRCC, signet ring cell adenocarcinoma; HR, hazard ratio.

In order to graphically appreciate overall differences in the distribution and HRs between adenocarcinomas and SRCCs by location, we have summarized these results by age group in Figure 3.

## 4. Discussion

Gastric and colorectal SRCCs account for over three-quarters of all SRCCs [3], and this study is the most comprehensive analysis to date of the histopathological characteristics by anatomic location for SRCCs arising in these two sites. There are multiple differences in the presentation and outcomes of SRCCs depending on age, sex, and location that require the case volume to deconvolute that is only possible through population registry level data.

Classically, the defining characteristics of SRCCs are higher stage at presentation and subsequently decreased survival. When comparing gastric cancer to colorectal cancer, the spread in behavior between the two histologies is not as dramatic (Figure 1 and Figure 2). Further, in early onset gastric cancer, outside of distant disease, patients with SRCCs statistically have similar outcomes compared to adenocarcinomas. This is partially explained by differences in the distribution of cancers between the two histologies. First, proximal gastric cancers typically have worse outcomes than distal gastric cancers [17]. We can demonstrate the same finding on both univariate and multivariable analyses for adenocarcinomas (distal to proximal HR 0.79, 0.93, respectively), and SRCCs (HR 0.75, 0.91, respectively) (Tables S2 and S3). Second, SRCCs occur more often in the distal stomach (33% vs 45% adenocarcinomas vs SRCCs). Finally, patients with early onset gastric cancer for both adenocarcinomas and SRCCs have longer cause specific survivals than late onset gastric cancer across all stages (Table S4). Therefore, a combination of all three factors help to explain the near overlapping survival curves in the <50-year-old group. However, in the late onset group (after 50 years of age), SRCC patients have worse outcomes across all stages, despite the increased prevalence of gastric cancer in the distal stomach attributed to the SRCC histotype. One of the largest studies ever conducted on the prognostic implication of gastric SRCC following radial gastrectomy with D2 lymph node dissection and R0 resection from South Korea demonstrated that early stage (Stage I) SRCC had a better prognosis than well-to-moderately differentiated adenocarcinoma after the adjustment for age, sex, and stage, but this trend reversed in advanced (Stage III) gastric cancer [18].

Comparing colorectal SRCCs to adenocarcinomas is much more straight forward. Outcomes are much worse for SRCC patients across all stages and grades, consistent with the literature [19], and SRCCs are more likely to arise in the right colon [20,21]. However, given that colorectal SRCCs comprise less than 1% of colorectal cancers, no studies have demonstrated a significant survival difference on the basis of location [19]. This study though by the use of population-level data is sufficiently powered to identify these differences. For adenocarcinomas, the mortality HR of rectal tumors relative to the rest of the colon on multivariable analysis is about 1.15. This HR however is only statistically significant for the ≥50-year-old group (1.17 (95% CI 1.14–1.20)) (<50-year-old group 1.06 (0.98–1.15)) (Table S6). This same pattern also holds for SRCCs, but the HR is higher at 1.53 (1.26–1.87) (Table S7). A recent meta-analysis concluded that increased colorectal SRCC pathogenesis can be correlated to both higher rates of BRAF mutations and microsatellite instability [22]. Further studies are needed to see if targeted therapies, including immunotherapy, may mitigate the dismal SRCC phenotype [23].

There is growing evidence that there may be linkages between gastric and colorectal SRCC risk for a subset of patients. Mutations in *CDH1* (e-cadherin) are linked to autosomal dominant hereditary diffuse gastric cancer, as well as increased lobular breast cancer in female carriers [24,25]. These gastric cancers present with the SRCC phenotype with a mean age of disease onset at 38 years, and account for about 1% of gastric cancer cases [26,27]. Carriers are recommended to have a prophylactic gastrectomy in early adult life, or otherwise undergo frequent endoscopic screenings to detect in situ cancer foci [24]. Despite multiple investigations to increase detection sensitivity, these foci are virtually invisible, and their detection essentially requires random biopsies [28,29,30,31]. The current gold standard protocol for biopsies is the Cambridge Protocol, where five random biopsies are taken from each of the prepyloric area, antrum, transition zone, body, fundus, and cardia [32,33]. However, we recently showed that this protocol does not increase detection rates significantly over truly random biopsies [34]. Given that SRCCs are enriched in the distal stomach, it is possible that detection rates might be increased by concentrating biopsy procurement in this area of the stomach. Further, we also showed that using SEER data that, in addition to the already known increased lobular breast cancer risk, patients with a primary gastric SRCC are at a three-fold increased risk of a colorectal SRCC compared to patients with conventional intestinal-type gastric adenocarcinomas, and that this risk is reciprocal among all three cancer types [34]. A subsequent study showed that *CDH1* germline variants are enriched in patients with colorectal SRCC, in addition to lobular breast cancer patients [35]. Taken together, these findings suggest the possibility of a signet ring cell syndrome underlying gastric SRCC, colorectal SRCC, and lobular breast cancer [34]. Further research is needed to substantiate this postulation, but we propose that patients presenting with any one of these cancers may warrant additional screening at the other sites.

This study does have several limitations. This is a retrospective study of a large population database where treatment variables are unfortunately limited to binary variables. Because the study covers cancers diagnosed over a nearly 30-year period and staging systems have undergone several evolutions over that time, it is necessary to label stages as broad categories to ensure the comparability of results and accumulate enough cases to allow for suitably powered analyses. Outside of a population-level database, this kind of investigation into sublocations within tumor sites would not be feasible given the overall rarity of SRCCs.

## 5. Conclusions

This study provides the most robust characterization of the demographic and histopathological composition of gastric and colorectal SRCCs by anatomical location. It illustrates systematically how SRCCs behave as a distinct histological subtype compared to conventional adenocarcinomas and consequently have distinct survival outcomes. Deeper investigations into the underpinnings of this tumor biology will be necessary to ultimately tailor screening, surveillance, and treatment approaches to improve prognosis.

## Figures and Tables

**Table 1:** Distribution of gastric cancer by localization.

Gastric Location	Adenocarcinoma	Signet Ring Cell
**All Sites**	44,239 (100)	9972 (100)
**Proximal**	29,519 (66.7)	5486 (55.0)
Cardia	21,828 (49.3)	2934 (29.4)
Fundus	2454 (5.5)	549 (5.5)
Body	5237 (11.8)	2003 (20.1)
**Distal**	14,720 (33.3)	4486 (45.0)
Antrum	12,783 (28.9)	3927 (39.4)
Pylorus	1937 (4.4)	559 (5.6)

*p* < 0.05 for all comparisons between adenocarcinomas and signet ring cell adenocarcinomas between proximal and distal stomach comparisons.

**Table 2:** Distribution of gastric cancer by localization, dichotomized by sex, and age groupings.

Gastric Location	Adenocarcinoma				Signet Ring Cell			
Gender	Male		Female		Male		Female	
Age (Years)	<50	≥50	<50	≥50	<50	≥50	<50	≥50
**All Sites**								
*N* (%)	2782 [9.2]	27,380 [90.8]	1086 [7.7]	12,991 [92.3]	1031 [18.8]	4466 [81.2]	984 [22.0]	3491 [78.0]
	(100)	(100)	(100)	(100)	(100)	(100)	(100)	(100)
Stage (%)								
In Situ	9 (0.3)	214 (0.8)	5 (0.5)	120 (0.9)	0 (0)	1 (<0.1)	0 (0)	2 (<0.1)
Localized	408 (14.7)	6711 (24.5)	181 (16.7)	3533 (27.2)	146 (14.2)	892 (20.0)	171 (17.4)	786 (22.5)
Regional	899 (32.3)	8308 (30.3)	367 (33.8)	3622 (27.9)	360 (34.9)	1585 (35.5)	309 (31.4)	1085 (31.1)
Distant	1354 (48.7)	9842 (35.9)	501 (46.1)	4033 (31.0)	480 (46.6)	1638 (36.7)	477 (48.5)	1254 (35.9)
Unstaged	114 (4.1)	2305 (8.4)	32 (2.9)	1683 (13.0)	4 (4.4)	350 (7.8)	27 (2.7)	364 (10.4)
Incidence	4.12 (3.95–4.29)	135 (133–137)	1.55 (1.45–1.66)	44.5 (43.7–45.3)	1.83 (1.72–1.95)	25.0 (24.3–25.7)	1.82 (1.71–1.94)	15.9 (15.5–16.4)
**Proximal**								
*N* (%)	2118 (76.1)	19,806 (72.3)	613 (56.4)	6982 (53.7)	564 (54.7)	2738 (61.3)	480 (48.8)	1707 (48.8)
Stage (%)								
In Situ	6 (0.3)	128 (0.6)	2 (0.3)	60 (0.9)	0 (0)	0 (0)	0 (0)	1 (0.1)
Localized	290 (13.7)	4697 (13.7)	102 (16.6)	1798 (25.8)	71 (12.6)	517 (18.9)	70 (14.6)	357 (21.0)
Regional	637 (30.1)	5684 (28.7)	184 (30.0)	1699 (24.3)	178 (31.6)	913 (33.3)	125 (26.0)	444 (26.1)
Distant	1090 (51.5)	7616 (38.5)	310 (50.6)	2493 (35.7)	290 (51.4)	1094 (40.0)	269 (56.0)	708 (41.5)
Unstaged	95 (4.5)	1681 (8.5)	15 (2.4)	932 (13.3)	25 (4.4)	214 (7.8)	16 (3.3)	194 (11.4)
Incidence	3.35 (3.20–3.50)	104 (103–106)	0.98 (0.90–1.07)	27.0 (26.4–27.6)	1.02 (0.93–1.10)	15.6 (15.1–16.2)	0.94 (0.86–1.03)	8.0 (7.7–8.3)
**Distal**								
*N* (%)	664 (23.9)	7574 (27.7)	473 (43.6)	6009 (46.3)	467 (45.3)	1728 (38.7)	504 (51.2)	1787 (51.2)
Stage (%)								
In Situ	1 (0.2)	86 (1.1)	3 (0.6)	60 (1.0)	0 (0)	1 (0.1)	0 (0)	1 (0.1)
Localized	118 (178)	2014 (26.6)	79 (16.7)	1735 (28.9)	75 (16.1)	375 (21.7)	101 (20.0)	429 (24.0)
Regional	262 (39.5)	2624 (34.6)	183 (38.7)	1923 (32.0)	182 (39.0)	672 (38.9)	184 (36.5)	641 (35.9)
Distant	264 (39.8)	2226 (29.4)	191 (40.4)	1540 (25.6)	190 (40.7)	544 (31.5)	208 (41.3)	546 (30.6)
Unstaged	19 (2.9)	624 (8.2)	17 (3.6)	751 (12.5)	20 (4.3)	136 (7.9)	11 (2.2)	170 (9.5)
Incidence	0.77 (0.70–0.85)	30.9 (30.1–31.7)	0.56 (0.50–0.63)	17.5 (17.0–18.0)	0.82 (0.74–0.90)	9.4 (9.0–9.9)	0.89 (0.80–0.96)	8.0 (7.6–8.3)

*p* < 0.05 for all comparisons between adenocarcinomas and signet ring cell adenocarcinomas. [ ] indicates percentages across the age groups within each sex; ( ) indicates percentages within each column. Incidence rates are expressed per 1 million.

**Table 3:** Derived univariate and multivariable Cox-proportional hazard ratios of mortality for gastric signet ring cell adenocarcinomas versus conventional adenocarcinomas.

Gastric Location	Signet Ring Cell vs. Adenocarcinoma (All Ages)		Signet Ring Cell vs. Adenocarcinoma (Age < 50)		Signet Ring Cell vs. Adenocarcinoma (Age ≥ 50)	
HR (95% CI)	Univariate	Multivariable	Univariate	Multivariable	Univariate	Multivariable
**All Sites**	1.14 (1.11–1.18)	1.11 (1.08–1.15)	1.05 (0.99–1.12)	1.05 (0.98–1.13)	1.17 (1.14–1.21)	1.12 (1.08–1.15)
**Proximal** Cardia Fundus Body	1.21 (1.17–1.25) 1.30 (1.24–1.36) 1.04 (0.93–1.17) 1.14 (1.07–1.22)	1.14 (1.10–1.18) 1.20 (1.14–1.26) 1.07 (0.95–1.20) 1.03 (0.96–1.11)	1.17 (1.08–1.28) 1.21 (1.07–1.36) 1.24 (0.96–1.61) 1.08 (0.92–1.27)	1.11 (1.01–1.22) 1.10 (0.97–1.25) 1.52 (1.12–2.06) 0.99 (0.83–1.17)	1.23 (1.18–1.27) 1.33 (1.27–1.40) 1.01 (0.89–1.15) 1.16 (1.07–1.24)	1.14 (1.09–1.19) 1.21 (1.15–1.28) 1.01 (0.89–1.16) 1.04 (0.96–1.13)
**Distal** Antrum Pylorus	1.14 (1.09–1.19) 1.17 (1.12–1.22) 0.98 (0.87–1.11)	1.08 (1.03–1.13) 1.09 (1.04–1.15) 0.99 (0.87–1.13)	1.03 (0.92–1.14) 1.01 (0.90–1.14) 1.10 (0.82–1.48)	0.99 (0.88–1.11) 0.99 (0.88–1.12) 0.87 (0.64–1.19)	1.18 (1.12–1.24) 1.21 (1.15–1.28) 0.98 (0.85–1.12)	1.10 (1.04–1.16) 1.11 (1.05–1.18) 0.98 (0.85–1.15)

*p* < 0.05 for all results unless confidence interval crosses 1. Multivariable adjustment corrected for sex, race, detection stage, grade differentiation, surgery, radiotherapy, and chemotherapy. HR, hazard ratios.

**Table 4:** Distribution of colorectal cancer by localization.

Colorectal Location	Adenocarcinoma	Signet Ring Cell
**All Sites**	393,879 (100)	6291 (100)
**Right Colon**	131,482 (33.4)	3481 (55.3)
Appendix	1761 (0.4)	669 (10.6)
Cecum	62,888 (16.0)	1480 (23.5)
Ascending Colon	51,752 (13.1)	1017 (16.2)
Hepatic Flexure	15,081 (3.8)	315 (5.0)
**Transverse Colon**	26,492 (6.7)	442 (7.0)
**Left**	116,632 (29.6)	1073 (17.1)
Splenic Flexure	10,985 (2.8)	134 (2.1)
Descending Colon	18,161 (4.6)	204 (3.2)
Sigmoid Colon	87,486 (22.2)	735 (11.7)
**Rectal**	119,273 (30.3)	1295 (20.6)
Rectosigmoid	37,657 (9.6)	327 (5.2)
Rectum	81,616 (20.7)	968 (15.4)

*p* < 0.05 for all comparisons between adenocarcinomas and signet ring cell adenocarcinomas between right colon, transverse colon, left colon, and rectal comparisons.

**Table 5:** Distribution of colorectal cancer by localization, dichotomized by sex, and age groupings.

Colorectal Location	Adenocarcinoma				Signet Ring Cell			
Sex	Male		Female		Male		Female	
Age (Years)	<50	≥50	<50	≥50	<50	≥50	<50	≥50
**All Sites**								
*N* (%)	22,579 [11.1]	181,541 [88.9]	19,470 [10.3]	170,289 [89.7]	764 [23.7]	2464 [76.3]	585 [19.1]	2478 [80.9]
	(100)	(100)	(100)	(100)	(100)	(100)	(100)	(100)
Stage								
In Situ	101 (0.4)	1292 (0.7)	95 (0.5)	1091 (0.6)	0 (0)	3 (0.1)	0 (0)	2 (0.1)
Localized	5263 (23.3)	58,867 (32.4)	4618 (23.7)	55,919 (32.8)	64 (8.4)	316 (12.8)	35 (6.0)	368 (14.9)
Regional	10,294 (45.6)	75,339 (41.5)	8895 (45.7)	72,384 (42.5)	342 (44.8)	1158 (47.0)	231 (39.5)	1086 (43.8)
Distant	6410 (28.4)	39,790 (21.9)	5545 (28.5)	34,201 (20.1)	341 (44.6)	926 (37.6)	310 (53.0)	944 (38.1)
Unstaged	511 (2.3)	6253 (3.4)	317 (1.6)	6694 (3.9)	17 (2.2)	61 (2.5)	9 (1.5)	78 (3.1)
Incidence	4.43 (4.38–4.49)	104.8 (104.4–105.3)	3.80 (3.75–3.85)	75.0 (74.7–75.3)	1.38 (1.29–1.49)	15.0 (14.5–15.6)	1.08 (1.00–1.17)	11.5 (11.1–12.0)
**Right**								
*N* (%)	4780 (21.2)	54,707 (30.1)	4171 (21.4)	67,824 (39.8)	281 (36.8)	1297 (52.6)	289 (49.4)	1614 (65.1)
Stage								
In Situ	23 (0.5)	401 (0.7)	19 (0.5)	417 (0.6)	0 (0)	1 (0.1)	0 (0)	1 (0.1)
Localized	1188 (24.9)	18,183 (33.2)	1010 (24.2)	23,069 (34.0)	26 (9.3)	163 (12.6)	20 (6.9)	247 (15.3)
Regional	2206 (46.2)	22,580 (41.3)	1787 (42.8)	28,555 (42.1)	113 (40.2)	603 (46.5)	103 (35.6)	702 (43.5)
Distant	1310 (27.4)	12,093 (22.1)	1308 (31.4)	13,618 (20.1)	138 (49.1)	511 (39.4)	163 (56.4)	633 (39.2)
Unstaged	53 (1.1)	1450 (2.7)	47 (1.1)	2165 (3.2)	4 (1.4)	19 (1.5)	3 (1.0)	31 (1.9)
Incidence	0.92 (0.90–0.95)	34.0 (33.7–34.2)	0.80 (0.78–0.82)	30.8 (30.6–31.0)	0.52 (0.46–0.59)	7.82 (7.44–8.23)	0.55 (0.49–0.62)	7.34 (7.03–7.70)
**Transverse**								
*N* (%)	1188 (5.3)	11,318 (6.2)	1062 (5.5)	12,924 (7.6)	32 (4.2)	158 (6.4)	28 (4.8)	224 (9.0)
Stage								
In Situ	2 (0.2)	64 (0.6)	6 (0.6)	64 (0.5)	0 (0)	0 (0)	0 (0)	0 (0)
Localized	285 (24.0)	3750 (33.1)	244 (23.0)	4224 (32.7)	2 (6.2)	17 (10.8)	2 (7.1)	45 (20.1)
Regional	588 (49.5)	4983 (44.0)	475 (44.7)	5899 (45.6)	18 (56.2)	89 (56.3)	16 (57.1)	112 (50.0)
Distant	297 (25.0)	2242 (19.8)	327 (30.8)	2360 (18.3)	12 (37.5)	47 (29.7)	10 (35.7)	56 (25.0)
Unstaged	16 (1.3)	279 (2.5)	10 (0.9)	10 (0.9)	0 (0)	5 (3.2)	0 (0)	11 (4.9)
Incidence	0.24 (0.23–0.25)	7.48 (7.36–7.60)	0.21 (0.20–0.22)	6.13 (6.03–6.22)	0.07 (0.05–0.10)	1.04 (0.90–1.20)	0.06 (0.04–0.08)	1.17 (1.04–1.31)
**Left**								
*N* (%)	6760 (29.9)	55,415 (30.5)	7205 (37.0)	47,252 (27.7)	190 (24.9)	425 (17.2)	127 (21.7)	331 (13.4)
Stage								
In Situ	39 (0.6)	453 (0.8)	36 (0.5)	325 (0.7)	0 (0)	0 (0)	0 (0)	0 (0)
Localized	1437 (21.3)	17,279 (31.2)	1593 (22.1)	14,719 (31.2)	16 (8.4)	51 (12.0)	1 (0.8)	37 (11.2)
Regional	2922 (43.2)	22,701 (41.0)	3250 (45.1)	20,191 (42.7)	72 (37.9)	169 (39.8)	48 (37.8)	129 (39.0)
Distant	2262 (33.5)	13,432 (24.2)	2261 (31.4)	10,464 (22.1)	99 (52.1)	196 (46.1)	77 (60.6)	152 (45.9)
Unstaged	100 (1.5)	1550 (2.8)	65 (0.9)	1553 (3.3)	3 (1.6)	9 (2.1)	1 (0.8)	12 (3.9)
Incidence	1.33 (1.30–1.36)	30.4 (30.2–30.6)	1.41 (1.38–1.44)	19.9 (19.7–20.0)	0.35 (0.30–0.40)	2.61 (2.40–2.85)	0.22 (0.18–0.26)	1.55 (1.40–1.71)
**Rectal**								
*N* (%)	9851 (43.6)	60,101 (33.1)	7032 (36.1)	42,289 (24.8)	261 (34.2)	584 (23.7)	141 (24.1)	309 (12.5)
Stage								
In Situ	37 (0.4)	374 (0.6)	34 (0.5)	285 (0.7)	0 (0)	2 (0.3)	0 (0)	1 (0.3)
Localized	2353 (23.9)	19,655 (32.7)	1771 (25.2)	13,907 (32.9)	20 (7.7)	85 (14.6)	12 (8.5)	39 (12.6)
Regional	4578 (46.5)	25,075 (41.7)	3383 (48.1)	17,739 (41.9)	139 (53.3)	297 (50.9)	64 (45.4)	143 (46.3)
Distant	2541 (25.8)	12,023 (20.0)	1649 (23.4)	7759 (18.3)	92 (35.2)	172 (29.5)	60 (42.6)	103 (33.3)
Unstaged	342 (3.5)	2974 (4.9)	195 (2.8)	2599 (6.1)	10 (3.8)	28 (4.8)	5 (3.5)	23 (7.4)
Incidence	1.94 (1.90–1.98)	32.9 (32.7–33.2)	1.39 (1.36–1.42)	18.2 (18.1–18.4)	0.44 (0.39–0.50)	3.52 (3.27–3.80)	0.25 (0.21–0.30)	1.46 (1.32–1.62)

*p* < 0.05 for all comparisons between adenocarcinomas and signet ring cell adenocarcinomas. [ ] indicates percentages across the age groups within each sex; ( ) indicates percentages within each column. Incidence rates are expressed per 100,000 for adenocarcinomas and per 1 million for signet ring cells.

**Table 6:** Derived univariate and multivariable Cox-proportional hazard ratios of mortality for colorectal signet ring cell adenocarcinomas versus conventional adenocarcinomas.

Colorectal Location	Signet Ring Cell vs. Adenocarcinoma (All Ages)		Signet Ring Cell vs. Adenocarcinoma (Age < 50)		Signet Ring Cell vs. Adenocarcinoma (Age ≥ 50)	
HR (95% CI)	Univariate	Multivariable	Univariate	Multivariable	Univariate	Multivariable
**All Sites**	2.39 (2.31–2.47)	1.55 (1.50–1.60)	2.99 (2.79–3.20)	1.78 (1.66–1.91)	2.30 (2.21–2.39)	1.46 (1.40–1.52)
**Transverse Colon**	2.01 (1.75–2.30)	1.38 (1.20–1.58)	2.47 (1.78–3.43)	1.90 (1.34–2.70)	1.94 (1.67–2.25)	1.29 (1.11–1.50)
**Right Colon**	2.17 (2.07–2.27)	1.28 (1.22–1.34)	2.66 (2.39–2.96)	1.37 (1.22–1.54)	2.10 (1.99–2.21)	1.24 (1.17–1.30)
Appendix	2.00 (1.77–2.26)	0.95 (0.82–1.09)	2.47 (1.95–3.12)	0.99 (0.74–1.30)	1.85 (1.60–2.14)	0.91 (0.77–1.08)
Cecum	2.28 (2.13–2.44)	1.35 (1.26–1.45)	2.80 (2.34–3.34)	1.45 (1.20–1.75)	2.24 (2.08–2.40)	1.33 (1.29–1.43)
Ascending Colon	1.85 (1.69–2.03)	1.24 (1.12–1.36)	2.34 (1.84–2.98)	1.54 (1.20–1.99)	1.79 (1.62–1.99)	1.19 (1.07–1.31)
Hepatic Flexure	2.23 (1.91–2.61)	1.83 (1.56–2.15)	2.40 (1.72–3.35)	1.81 (1.25–2.63)	2.18 (1.83–2.60)	1.73 (1.44–2.07)
**Left**	2.88 (2.67–3.11)	1.78 (1.64–1.92)	3.42 (2.98–3.92)	2.00 (1.73–2.31)	2.75 (2.51–3.03)	1.60 (1.45–1.76)
Splenic Flexure	1.86 (1.46–2.36)	1.45 (1.14–1.86)	2.04 (1.33–3.13)	1.25 (0.79–1.98)	1.89 (1.41–2.52)	1.36 (1.01–1.82)
Descending Colon	2.88 (2.42–3.42)	1.84 (1.54–2.20)	3.97 (3.01–5.24)	2.05 (1.52–2.78)	2.55 (2.03–3.20)	1.56 (1.24–1.97)
Sigmoid Colon	3.12 (2.84–3.42)	1.82 (1.65–2.00)	3.67 (3.10–4.35)	2.11 (1.78–2.53)	2.99 (2.68–3.34)	1.66 (1.49–1.86)
**Rectal**	2.70 (2.53–2.90)	2.10 (1.96–2.25)	3.10 (2.74–3.50)	1.90 (1.67–2.16)	2.73 (2.51–2.96)	2.01 (1.85–2.19)
Rectosigmoid	2.98 (2.60–3.40)	2.02 (1.77–2.32)	3.32 (2.58–4.28)	1.70 (1.30–2.21)	2.96 (2.53–3.47)	1.98 (1.69–2.33)
Rectum	2.61 (2.41–2.82)	2.11 (1.94–2.27)	3.05 (2.65–3.51)	1.95 (1.68–2.27)	2.63 (2.39–2.90)	2.00 (1.81–2.20)

*p* < 0.05 for all results, unless confidence interval crosses 1. Multivariable adjustment corrected for sex, race, detection stage, grade differentiation, surgery, radiotherapy, and chemotherapy. HR, hazard ratios.

## Data Availability

Data release from the SEER database (https://seer.cancer.gov, accessed 15 September 2022).

## Supplementary Materials

The following supporting information can be downloaded at: https://www.mdpi.com/article/10.3390/cancers15030714/s1. Table S1: Definitions of organ localization by ICD-O-3 codes within this study; Table S2: Derived univariate and multivariable Cox-proportional hazard ratios of mortality for gastric adenocarcinomas; Table S3: Derived univariate and multivariable Cox-proportional hazard ratios of mortality for gastric SRCCs; Table S4: Cause-specific survival of gastric conventional adenocarcinomas and SRCCs by age group and site; Table S5: Relative survival of gastric conventional adenocarcinomas and SRCCs by age group and site; Table S6: Derived univariate and multivariable Cox-proportional hazard ratios of mortality for colorectal adenocarcinomas; Table S7: Derived univariate and multivariable Cox-proportional hazard ratios of mortality for colorectal SRCCs; Table S8: Cause-specific survival of colorectal conventional adenocarcinomas and SRCCs by age group and site; Table S9: Relative survival of colorectal conventional adenocarcinomas and SRCCs by age group and site.
