# Supplementary material for: Effects of Tumor Localization, Age, and Stage on the Outcomes of Gastric and Colorectal Signet Ring Cell Adenocarcinomas
Source: Cancers (Basel). 2023 Jan 24;15(3):714. doi: 10.3390/cancers15030714 (PMC9913295; doi:10.3390/cancers15030714)
Supplement: Supplementary file 1 [file cancers-15-00714-s001.zip › cancers-2114703-supplementary.pdf]

cancers

Supplementary Material

Effects of Tumor Localization, Age, and Stage on the Outcomes of Gastric and Colorectal Signet Ring Cell Adenocarcinomas

Table S1. Definitions of organ localization by ICD-O-3 codes within this study.

Gastric*	Colorectal**
**Proximal Stomach**<br>Cardia – C16.0<br>Fundus – C16.1<br>Body – C16.2	**Right Colon**<br>Cecum – C18.0<br>Appendix – C18.1<br>Ascending Colon – C18.2<br>Hepatic Flexure of Colon – C18.3
**Distal Stomach**<br>Antrum – C16.3<br>Pylorus – C16.4	**Transverse Colon** – C18.4
	**Left Colon**<br>Splenic Flexure of Colon – C18.5<br>Descending Colon – C18.6<br>Sigmoid Colon – C18.7
	**Rectal**<br>Rectosigmoid junction – C19.9<br>Rectum – C20.9

*Excluded gastric codes are C16.5 (lesser curvature of stomach, NOS (not otherwise specified) not classifiable to C16.1-C16.4), C16.6 (greater curvature of stomach, NOS, not classifiable to C16.0-C16.4), C16.8 (overlapping lesion of stomach), and C16.9 (stomach, NOS).
**Excluded colorectal codes are C18.8 (overlapping lesion of colon), C19.9 (colon, NOS).

Table S2. Derived univariate and multivariable Cox-proportional hazard ratios of mortality for gastric adenocarcinomas.

Gastric Location	Adenocarcinoma (All Ages)		Adenocarcinoma (Age <50)		Adenocarcinoma (Age ≥50)	
**HR (95% CI)**	**Univariate**	**Multivariable**	**Univariate**	**Multivariable**	**Univariate**	**Multivariable**
**Proximal**	1.00 (Reference)	1.00 (Reference)	1.00 (Reference)	1.00 (Reference)	1.00 (Reference)	1.00 (Reference)
**Distal**	0.79 (0.77-0.81)	0.93 (0.90-0.95)	0.82 (0.75-0.90)	1.01 (0.92-1.11)	0.79 (0.77-0.81)	0.91 (0.88-0.94)
**Cardia**	1.00 (Reference)	1.00 (Reference)	1.00 (Reference)	1.00 (Reference)	1.00 (Reference)	1.00 (Reference)
**Fundus**	1.20 (1.14-1.27)	1.10 (1.04-1.15)	1.11 (0.94-1.32)	1.02 (0.86-1.21)	1.21 (1.15-1.28)	1.10 (1.03-1.16)
**Body**	0.95 (0.92-0.99)	0.96 (0.92-1.00)	1.04 (0.91-1.18)	1.03 (0.90-1.19)	0.95 (0.91-0.99)	0.94 (0.90-0.99)
**Antrum**	0.79 (0.77-0.81)	0.91 (0.88-0.94)	0.85 (0.77-0.94)	0.99 (0.89-1.11)	0.79 (0.76-0.81)	0.89 (0.86-0.92)
**Pylorus**	0.82 (0.77-0.88)	0.91 (0.85-0.97)	0.73 (0.59-0.90)	0.93 (0.74-1.17)	0.83 (0.78-0.89)	0.90 (0.84-0.96)

$p < 0.05$ for all results unless confidence interval crosses 1. Multivariable adjustment corrected for gender, race, detection stage, grade differentiation, surgery, radiotherapy, and chemotherapy. HR, hazard ratios.

Table S3. Derived univariate and multivariable Cox-proportional hazard ratios of mortality for gastric SRCCs.

Gastric Location	Signet Ring Cell (All Ages)		Signet Ring Cell (Age <50)		Signet Ring Cell (Age ≥50)	
**HR (95% CI)**	**Univariate**	**Multivariable**	**Univariate**	**Multivariable**	**Univariate**	**Multivariable**
**Proximal**	1.00 (Reference)	1.00 (Reference)	1.00 (Reference)	1.00 (Reference)	1.00 (Reference)	1.00 (Reference)
**Distal**	0.75 (0.71-0.79)	0.91 (0.86-0.96)	0.72 (0.65-0.80)	0.90 (0.80-1.01)	0.76 (0.72-0.81)	0.91 (0.86-0.96)
**Cardia**	1.00 (Reference)	1.00 (Reference)	1.00 (Reference)	1.00 (Reference)	1.00 (Reference)	1.00 (Reference)
**Fundus**	0.97 (0.87-1.08)	0.96 (0.86-1.09)	1.18 (0.94-1.49)	1.31 (1.01-1.69)	0.92 (0.81-1.04)	0.90 (0.79-1.03)
**Body**	0.85 (0.79-0.91)	0.86 (0.80-0.93)	0.93 (0.80-1.09)	0.93 (0.78-1.10)	0.84 (0.77-0.90)	0.93 (0.79-1.10)
**Antrum**	0.71 (0.67-0.76)	0.86 (0.80-0.93)	0.72 (0.62-0.82)	0.93 (0.80-1.07)	0.72 (0.68-0.77)	0.85 (0.79-0.91)
**Pylorus**	0.61 (0.54-0.69)	0.77 (0.67-0.87)	0.68 (0.53-0.86)	0.94 (0.72-1.23)	0.60 (0.52-0.69)	0.73 (0.63-0.85)

$p < 0.05$ for all results unless confidence interval crosses 1. Multivariable adjustment corrected for gender, race, detection stage, grade differentiation, surgery, radiotherapy, and chemotherapy. HR, hazard ratios.

Table S4. Cause-specific survival of gastric conventional adenocarcinomas and SRCCs by age group and site.

Gastric Location	All Patients		Age < 50 years		Age ≥ 50 years	
**Cause-Specific Survival % (95% CI)**	**Adenocarcinoma**	**Signet Ring Cell**	**Adenocarcinoma**	**Signet Ring Cell**	**Adenocarcinoma**	**Signet Ring Cell**
**All Sites**						
**Localized**						
1-year	76.8 (75.7-77.8)	73.8 (71.6-75.9)	87.8 (83.9-90.8)	86.5 (81.5-90.2)	76.1 (75.0-77.2)	71.5 (69.0-73.8)
2-year	66.2 (65.0-67.3)	62.6 (60.2-65.0)	82.0 (77.6-85.7)	77.4 (71.6-82.1)	65.2 (64.0-66.4)	59.9 (57.2-62.5)
5-year	54.3 (53.0-55.5)	51.0 (48.4-53.6)	71.4 (66.3-75.8)	66.9 (60.5-72.5)	53.2 (51.9-54.5)	48.1 (45.2-50.8)
10-year	48.8 (47.4-50.2)	46.1 (43.4-48.8)	67.5 (62.1-72.4)	60.9 (53.9-67.2)	47.6 (46.2-49.0)	43.4 (40.4-46.3)
Median (months)	96.8	69.2	-	-	85.1	51.7
**Regional**						
1-year	66.6 (65.5-67.7)	66.0 (64.1-67.9)	75.6 (72.2-78.6)	79.5 (75.6-82.8)	65.7 (64.5-66.8)	62.7 (60.6-64.8)
2-year	46.2 (45.0-47.3)	44.9 (42.9-46.8)	54.4 (50.6-58.0)	58.1 (53.5-62.4)	45.3 (44.1-46.5)	41.6 (39.4-43.8)
5-year	28.6 (27.5-29.7)	25.4 (23.6-27.2)	36.5 (32.8-40.1)	33.3 (29.0-37.6)	27.7 (26.6-28.9)	23.4 (21.5-25.4)
10-year	22.6 (21.5-23.7)	19.3 (17.6-21.1)	29.3 (25.6-33.0)	24.9 (20.8-29.2)	21.9 (20.7-23.0)	18.0 (16.1-19.9)
Median (months)	20.9	20.1	27.7	31.5	20.3	17.9
**Distant**						
1-year	29.6 (28.7-30.5)	25.7 (24.1-27.3)	34.6 (31.9-37.3)	28.5 (25.2-31.8)	28.9 (28.0-29.9)	24.8 (23.0-26.6)
2-year	13.2 (12.6-13.9)	10.0 (9.0-11.2)	16.2 (14.4-18.3)	10.5 (8.4-13.0)	12.8 (12.1-13.6)	9.9 (8.7-11.2)
5-year	5.0 (4.6-5.5)	2.9 (2.3-3.6)	5.2 (4.0-6.6)	2.8 (1.7-4.3)	5.0 (4.6-5.5)	3.0 (2.3-3.8)
10-year	3.8 (3.4-4.3)	2.3 (1.7-2.9)	3.0 (2.8-5.2)	2.6 (1.6-4.1)	3.8 (3.4-4.3)	2.2 (1.6-3.0)
Median (months)	6.0	5.9	8.4	7.0	5.7	5.5
**Proximal**						
**Localized**						
1-year	76.1 (74.9-77.3)	70.0 (66.8-73.1)	87.1 (82.4-90.6)	83.9 (75.7-89.6)	75.5 (74.2-76.7)	67.8 (64.2-71.1)
2-year	64.0 (626-65.4)	56.3 (52.8-59.7)	80.2 (74.8-84.5)	71.3 (61.9-78.8)	63.0 (61.6-64.5)	53.9 (50.1-57.5)
5-year	51.5 (50.0-53.0)	43.1 (39.5-46.6)	70.3 (64.3-75.5)	57.7 (47.8-66.4)	50.3 (48.7-51.9)	40.7 (36.9-44.5)
10-year	45.7 (44.1-47.3)	37.7 (34.1-41.4)	66.3 (59.8-71.9)	53.7 (43.562.9)	44.3 (42.6-46.0)	35.1 (31.2-39.0)
Median (months)	69.9	35.4	-	-	61.0	31.6
**Regional**						
1-year	68.2 (66.9-69.5)	62.9 (60.2-65.5)	77.1 (73.2-80.5)	77.3 (71.2-82.2)	67.2 (65.8-68.6)	59.8 (56.7-62.7)
2-year	46.9 (45.5-48.3)	41.1 (38.3-43.8)	54.8 (50.3-59.0)	55.8 (49.0-62.1)	46.0 (44.5-47.5)	37.8 (34.8-40.8)
5-year	27.9 (26.6-29.2)	21.6 (19.3-24.1)	35.0 (30.8-39.3)	28.9 (22.9-35.1)	27.1 (25.7-28.5)	20.0 (17.5-22.7)
10-year	21.6 (20.3-23.0)	16.8 (14.6-19.3)	27.2 (23.0-31.5)	21.7 (16.1-27.9)	21.0 (19.6-22.4)	15.8 (13.4-18.5)
Median (months)	21.7	17.6	27.4	29.4	21.0	16.2
**Distant**						
1-year	30.7 (29.7-31.7)	25.1 (23.2-27.1)	36.5 (33.4-39.5)	27.7 (23.5-32.0)	29.9 (28.8-31.0)	24.4 (22.2-26.6)
2-year	13.6 (12.8-14.4)	8.9 (7.7-10.3)	17.2 (14.8-19.7)	8.7 (6.2-11.8)	13.1 (12.3-13.9)	9.0 (7.5-10.6)
5-year	5.0 (4.5-5.6)	2.5 (1.8-3.4)	5.5 (4.1-7.2)	2.2 (1.0-4.0)	5.0 (4.4-5.5)	2.6 (1.8-3.7)
10-year	3.7 (3.2-4.2)	2.1 (1.4-2.9)	4.0 (2.8-5.6)	2.2 (1.0-4.0)	3.6 (3.1-4.2)	2.1 (1.4-3.1)
Median (months)	6.5	5.9	9.0	7.0	6.2	5.6
**Distal**						
**Localized**						
1-year	78.3 (76.4-80.1)	77.8 (74.7-80.6)	89.9 (81.4-94.6)	88.6 (81.9-93.0)	77.7 (75.8-79.6)	75.6 (72.0-78.7)
2-year	71.3 (69.2-73.3)	69.3 (65.9-72.5)	87.6 (78.7-92.9)	82.5 (74.9-88.0)	70.5 (68.3-72.6)	66.6 (62.7-70.1)
5-year	60.9 (58.6-63.2)	59.4 (55.8-62.9)	74.5 (63.9-82.5)	74.5 (66.0-81.2)	60.3 (57.9-62.6)	56.2 (52.1-60.1)
10-year	56.3 (53.8-58.7)	54.9 (50.9-58.7)	71.3 (60.2-79.9)	67.0 (57.2-75.1)	55.5 (52.9-58.0)	52.4 (48.0-56.6)
Median (months)	-	-	-	-	-	-
**Regional**						
1-year	63.0 (61.0-65.0)	69.2 (66.6-71.7)	71.5 (64.5-77.3)	81.4 (76.1-85.6)	62.2 (60.1-64.3)	65.9 (62.8-68.8)
2-year	44.6 (42.5-46.7)	48.8 (45.9-51.6)	53.4 (46.1-60.2)	60.0 (53.8-65.7)	43.8 (41.6-45.9)	45.7 (42.5-48.9)
5-year	30.1 (42.5-46.7)	29.2 (26.6-31.9)	40.2 (33.1-47.2)	37.0 (31.0-42.9)	29.1 (27.1-31.2)	27.1 (24.2-30.0)
10-year	24.6 (22.7-26.6)	22.0 (1.4-24.6)	34.5 (27.5-41.6)	27.6 (21.9-33.6)	23.6 (21.6-25.7)	20.4 (17.6-23.4)
Median (months)	19.3	23.1	28.4	34.4	18.9	20.3
**Distant**						
1-year	25.9 (24.2-27.7)	26.7 (24.1-29.4)	27.5 (22.1-33.2)	29.7 (24.6-35.0)	25.8 (23.9-27.7)	25.5 (22.6-28.6)
2-year	12.1 (10.8-13.4)	12.0 (10.1-14.0)	12.3 (8.5-16.8)	13.1 (9.5-17.4)	12.0 (10.7-13.5)	11.5 (9.4-13.9)
5-year	5.1 (4.2-6.1)	3.6 (2.5-4.9)	4.0 (2.0-7.2)	3.6 (1.8-6.5)	5.3 (4.3-6.4)	3.5 (2.3-5.1)
10-year	4.3 (3.4-5.3)	2.6 (1.7-3.8)	3.4 (1.5-6.5)	3.2 (1.5-6.0)	4.4 (3.5-5.5)	2.4 (1.4-3.8)
Median (months)	4.5	6.0	6.5	7.1	4.3	5.4

CI, confidence interval; Proximal includes cardia, fundus, and body; Distal includes antrum and pylorus

cancers

MDPI

Table S5. Relative survival of gastric conventional adenocarcinomas and SRCCs by age group and site.

Gastric Location	All Patients		Age < 50 years		Age ≥50 years	
**Relative Survival % (95% CI)**	**Adenocarcinoma**	**Signet Ring Cell**	**Adenocarcinoma**	**Signet Ring Cell**	**Adenocarcinoma**	**Signet Ring Cell**
**All Sites**						
**Localized**						
1-year	74.0 (72.9-75.1)	71.3 (69.0-73.5)	86.9 (82.9-90.0)	86.9 (82.0-90.6)	73.3 (72.1-74.4)	68.5 (65.9-71.0)
2-year	63.0 (61.8-64.3)	60.2 (57.7-62.7)	80.4 (75.8-84.2)	77.3 (71.5-82.1)	62.0 (60.7-63.3)	57.1 (54.3-59.8)
5-year	50.4 (49.0-51.8)	48.4 (45.7-51.1)	68.2 (62.9-72.8)	66.5 (60.1-72.2)	49.4 (47.9-50.8)	45.1 (42.1-48.1)
10-year	42.6 (40.8-44.3)	42.5 (39.3-45.6)	62.0 (56.1-67.3)	60.1 (52.9-66.6)	41.4 (39.5-43.2)	39.2 (35.6-42.7)
Median (months)	61.8	52.7	-	-	56.8	38.9
**Regional**						
1-year	65.3 (64.2-66.4)	65.0 (63.1-66.9)	74.7 (71.3-77.8)	78.7 (74.8-82.0)	64.3 (63.1-65.5)	61.7 (59.5-63.8)
2-year	44.6 (43.4-45.8)	43.7 (41.7-45.7)	53.0 (49.3-56.6)	56.7 (52.2-60.9)	43.7 (42.4-44.9)	40.5 (38.3-42.7)
5-year	27.1 (26.0-28.2)	24.2 (22.4-26.0)	34.8 (31.2-38.4)	31.7 (27.6-35.9)	26.3 (25.1-27.5)	22.4 (20.4-24.3)
10-year	20.7 (19.5-22.0)	17.7 (16.0-19.6)	26.7 (23.2-30.4)	22.5 (18.6-26.6)	20.1 (18.8-21.4)	16.6 (14.6-18.7)
Median (months)	19.9	19.1	26.6	30.2	19.2	17.3
**Distant**						
1-year	28.7 (27.8-29.5)	25.4 (23.8-26.9)	34.2 (31.5-36.8)	28.2 (25.0-31.4)	27.9 (27.0-28.9)	24.4 (22.7-26.2)
2-year	12.6 (12.0-13.3)	9.6 (8.5-10.7)	15.9 (13.9-18.0)	9.9 (7.9-12.2)	12.2 (11.5-12.9)	9.5 (8.3-10.7)
5-year	4.6 (4.2-5.1)	2.8 (2.2-3.5)	4.9 (3.8-6.3)	2.8 (1.8-4.3)	4.6 (4.1-5.1)	2.8 (2.2-3.6)
10-year	3.5 (3.0-3.9)	2.0 (1.5-2.7)	3.7 (2.7-5.0)	2.5 (1.5-3.9)	3.4 (3.0-3.9)	1.9 (1.3-2.7)
Median (months)	5.8	5.8	8.3	7.0	5.5	5.3
**Proximal**						
**Localized**						
1-year	73.4 (72.0-74.7)	67.4 (64.0-70.5)	85.9 (81.1-89.5)	84.4 (76.2-89.9)	72.6 (71.2-74.0)	64.7 (61.0-68.2)
2-year	60.8 (59.3-62.3)	54.2 (50.6-57.6)	77.9 (72.4-82.5)	72.1 (62.8-79.5)	59.7 (58.2-61.3)	51.3 (47.4-55.1)
5-year	47.5 (45.9-49.2)	40.3 (36.6-43.9)	66.5 (60.3-71.9)	58.0 (48.0-66.8)	46.3 (44.6-48.1)	37.4 (33.5-41.4)
10-year	39.6 (37.6-41.7)	34.1 (30.0-38.3)	60.5 (53.8-66.6)	54.5 (44.1-63.8)	38.2 (36.1-40.3)	30.8 (26.3-35.3)
Median (months)	48.3	32.0	-	-	44.7	26.0
**Regional**						
1-year	66.8 (65.5-68.1)	62.6 (59.9-65.3)	77.0 (73.1-80.4)	77.2 (71.2-82.1)	65.7 (64.3-67.1)	59.4 (56.3-62.4)
2-year	45.1 (43.7-46.6)	39.9 (37.2-42.7)	53.9 (49.4-58.1)	54.2 (47.5-60.4)	44.2 (42.7-45.7)	36.8 (33.7-39.8)
5-year	26.2 (24.9-27.5)	20.9 (18.5-23.3)	33.6 (29.4-37.8)	27.3 (21.5-33.3)	25.3 (23.9-26.7)	19.5 (16.9-22.1)
10-year	19.5 (18.1-21.0)	15.1 (12.8-17.6)	24.7 (20.6-29.0)	18.6 (13.4-24.5)	19.0 (17.4-20.5)	14.3 (11.7-17.1)
Median (months)	20.5	17.0	26.7	28.5	19.9	15.9
**Distant**						
1-year	29.7 (28.7-30.7)	24.7 (22.8-26.7)	35.9 (32.9-38.9)	27.2 (23.2-31.4)	28.9 (27.8-29.9)	24.0 (21.8-26.2)
2-year	13.0 (12.2-13.7)	8.4 (7.2-9.7)	16.8 (32.9-38.9)	8.3 (5.9-11.2)	12.4 (11.7-13.2)	8.4 (7.0-9.9)
5-year	4.7 (4.2-5.2)	2.4 (1.8-3.3)	5.2 (3.9-6.8)	2.3 (1.1-4.1)	4.6 (4.1-5.1)	2.5 (1.7-3.5)
10-year	3.3 (2.9-3.9)	2.1 (1.4-2.9)	3.9 (2.7-5.4)	2.3 (1.1-4.1)	3.3 (2.7-3.9)	2.0 (1.3-3.0)
Median (months)	6.3	5.8	8.9	7.0	5.9	5.5
**Distal**						
**Localized**						
1-year	75.6 (73.4-77.5)	75.6 (72.2-78.5)	90.1 (81.6-94.8)	89.1 (82.4-93.3)	74.9 (72.7-76.9)	72.8 (69.0-76.2)
2-year	68.5 (66.1-70.7)	66.7 (63.1-70.1)	88.0 (78.9-93.3)	81.7 (74.0-87.3)	67.6 (65.1-69.9)	63.6 (59.5-67.4)
5-year	57.3 (54.5-60.0)	57.1 (53.1-60.9)	73.3 (62.3-81.6)	73.5 (64.9-80.3)	56.6 (53.7-59.3)	53.6 (49.1-57.9)
10-year	49.7 (46.0-53.2)	57.1 (46.2-55.8)	66.1 (53.4-76.1)	64.5 (54.2-73.0)	48.8 (45.0-52.4)	48.3 (42.8-53.7)
Median (months)	117	-	-	-	111	107
**Regional**						
1-year	62.0 (59.9-64.0)	67.4 (64.7-70.0)	68.9 (62.0-74.9)	80.0 (74.6-84.3)	61.3 (59.1-63.4)	64.0 (60.9-67.0)
2-year	43.4 (41.3-45.5)	47.5 (44.6-50.3)	50.9 (43.8-57.6)	58.8 (52.7-64.5)	42.7 (40.4-44.9)	44.4 (41.2-47.6)
5-year	29.2 (27.2-31.3)	27.5 (24.9-30.2)	37.8 (31.0-44.6)	35.5 (29.7-41.3)	28.4 (26.2-30.6)	25.4 (22.5-28.4)
10-year	23.3 (21.0-25.7)	20.3 (17.7-23.0)	31.5 (24.8-38.5)	25.7 (20.2-31.6)	22.5 (20.0-25.0)	18.8 (15.9-21.9)
Median (months)	18.6	21.7	26.4	33.3	18.3	19.3
**Distant**						
1-year	25.3 (23.5-27.0)	26.4 (23.9-29.0)	27.6 (22.3-33.2)	29.5 (34.5-34.7)	25.0 (23.2-26.9)	25.2 (22.3-28.2)
2-year	11.6 (10.3-12.9)	11.6 (9.8-13.6)	12.7 (8.9-17.1)	12.3 (8.8-16.3)	11.5 (10.1-12.9)	11.3 (9.2-13.6)
5-year	4.4 (3.6-5.4)	3.5 (2.5-4.8)	3.8 (1.9-6.8)	3.7 (1.9-6.4)	4.5 (3.6-5.5)	3.4 (2.2-5.0)
10-year	3.8 (2.9-4.8)	1.9 (1.0-3.2)	3.2 (1.4-6.2)	2.7 (1.1-5.4)	3.8 (2.9-4.9)	1.7 (0.8-3.2)
Median (months)	4.4	5.7	6.3	7.1	4.1	5.0

CI, confidence interval; Proximal includes cardia, fundus, and body; Distal includes antrum and pylorus

Table S6. Derived univariate and multivariable Cox-proportional hazard ratios of mortality for colorectal adenocarcinomas.

**Colorectal Location**	**Adenocarcinoma (All Ages)**		**Adenocarcinoma (Age <50)**		**Adenocarcinoma (Age ≥50)**	
**HR (95% CI)**	**Univariate**	**Multivariable**	**Univariate**	**Multivariable**	**Univariate**	**Multivariable**
**Transverse Colon**	1.00 (Reference)	1.00 (Reference)	1.00 (Reference)	1.00 (Reference)	1.00 (Reference)	1.00 (Reference)
**Right Colon**	1.07 (1.05-1.10)	1.01 (0.99-1.04)	1.02 (0.95-1.11)	1.03 (0.95-1.12)	1.08 (1.05-1.10)	1.01 (0.99-1.04)
Appendix	1.31 (1.21-1.42)	1.16 (1.07-1.26)	1.25 (1.05-1.49)	1.40 (1.16-1.67)	1.34 (1.23-1.47)	1.12 (1.03-1.23)
Cecum	1.18 (1.14-1.20)	1.05 (1.02-1.07)	1.07 (0.98-1.16)	1.00 (0.91-1.09)	1.18 (1.15-1.21)	1.05 (1.03-1.08)
Ascending Colon	0.95 (0.93-0.98)	0.96 (0.93-0.99)	0.93 (0.85-1.02)	1.02 (0.93-1.12)	0.95 (0.93-0.98)	0.96 (0.93-0.98)
Hepatic Flexure	1.07 (1.03-1.10)	1.03 (0.99-1.06)	1.10 (0.97-1.24)	1.10 (0.98-1.24)	1.06 (1.03-1.10)	1.02 (0.98-1.06)
**Left**	1.02 (1.00-1.05)	0.96 (0.94-0.98)	1.03 (0.95-1.11)	0.94 (0.87-1.02)	1.03 (1.01-1.06)	0.97 (0.95-1.00)
Splenic Flexure	1.10 (1.06-1.15)	1.05 (1.01-1.10)	1.00 (0.89-1.13)	1.02 (0.90-1.15)	1.12 (1.07-1.16)	1.05 (1.01-1.10)
Descending Colon	1.02 (0.99-1.06)	0.96 (0.93-0.99)	0.98 (0.89-1.09)	0.91 (0.82-1.01)	1.03 (0.99-1.06)	0.97 (0.93-1.00)
Sigmoid Colon	1.02 (1.00-1.05)	0.94 (0.92-0.97)	1.05 (0.97-1.13)	0.94 (0.87-1.02)	1.02 (1.00-1.05)	0.96 0.93-0.98)
**Rectal**	1.14 (1.12-1.17)	1.15 (1.12-1.18)	1.02 (0.95-1.10)	1.06 (0.98-1.15)	1.17 (1.14-1.20)	1.17 (1.14-1.20)
Rectosigmoid	1.10 (1.07-1.13)	1.05 (1.02-1.08)	1.05 (0.97-1.15)	1.00 (0.91-1.10)	1.11 (1.08-1.14)	1.07 (1.04-1.10)
Rectum	1.16 (1.14-1.19)	1.21 (1.18-1.24)	1.01 (0.93-1.09)	1.10 (1.00-1.21)	1.20 (1.17-.23)	1.24 (1.20-1.27)

$p < 0.05$ for all results unless confidence interval crosses 1. Multivariable adjustment corrected for gender, race, detection stage, grade differentiation, surgery, radiotherapy, and chemotherapy. HR, hazard ratios.

Table S7. Derived univariate and multivariable Cox-proportional hazard ratios of mortality for colorectal SRCCs.

**Colorectal Location**	**Signet Ring Cell (All Ages)**		**Signet Ring Cell (Age <50)**		**Signet Ring Cell (Age ≥50)**	
**HR (95% CI)**	**Univariate**	**Multivariable**	**Univariate**	**Multivariable**	**Univariate**	**Multivariable**
**Transverse Colon**	1.00 (Reference)	1.00 (Reference)	1.00 (Reference)	1.00 (Reference)	1.00 (Reference)	1.00 (Reference)
**Right Colon**	1.16 (1.01-1.33)	0.94 (0.81-1.09)	1.12 (0.80-1.56)	0.91 (0.64-1.30)	1.16 (0.99-1.36)	0.96 (0.82-1.12)
Appendix	1.25 (1.06-1.48)	0.81 (0.67-0.99)	1.23 (0.86-1.77)	0.76 (0.47-1.27)	1.22 (1.01-1.47)	0.84 (0.67-1.06)
Cecum	1.33 (1.14-1.54)	1.03 (0.89-1.20)	1.20 (0.84-1.72)	0.94 (0.62-1.43)	1.35 (1.15-1.59)	1.07 (0.91-1.27)
Ascending Colon	0.88 (0.75-1.04)	0.88 (0.75-1.04)	0.90 (0.61-1.34)	0.86 (0.56-1.32)	0.89 (0.74-1.06)	0.89 (0.74-1.06)
Hepatic Flexure	1.20 (0.98-1.47)	1.29 (1.04-1.58)	1.12 (0.71-1.76)	1.04 (0.65-1.68)	1.21 (0.96-1.52)	1.31 (1.04-1.65)
**Left**	1.39 (1.19-1.62)	1.09 (0.93-1.28)	1.28 (0.91-1.81)	0.97 (0.68-1.39)	1.38 (1.16-1.64)	1.10 (0.92-1.33)
Splenic Flexure	1.00 (0.76-1.31)	0.97 (0.73-1.29)	0.80 (0.47-1.36)	0.58 (0.33-1.03)	1.06 (0.77-1.47)	1.07 (0.76-1.49)
Descending Colon	1.37 (1.10-1.71)	1.13 (0.90-1.43)	1.41 (0.93-2.15)	1.11 (0.71-1.72)	1.30 (0.99-1.70)	1.16 (0.87-1.54)
Sigmoid Colon	1.47 (1.25-1.73)	1.09 (0.92-1.30)	1.37 (0.96-1.97)	0.97 (0.66-1.42)	1.47 (1.22-1.77)	1.13 (0.93-1.38)
**Rectal**	1.43 (1.23-1.66)	1.45 (1.22-1.73)	1.14 (0.81-1.60)	1.10 (0.75-1.62)	1.52 (1.28-1.80)	1.53 (1.26-1.87)
Rectosigmoid	1.50 (1.24-1.81)	1.23 (0.99-1.53)	1.27 (0.84-1.90)	1.03 (0.64-1.65)	1.56 (1.26-1.94)	1.30 (1.01-1.68)
Rectum	1.40 (1.20-1.64)	1.51 (1.24-1.85)	1.11 (0.79-1.58)	1.16 (0.76-1.77)	1.50 (1.26-1.79)	1.60 (1.28-2.02)

$p < 0.05$ for all results unless confidence interval crosses 1. Multivariable adjustment corrected for gender, race, detection stage, grade differentiation, surgery, radiotherapy, and chemotherapy. HR, hazard ratios.

Table S8. Cause-specific survival of colorectal conventional adenocarcinomas and SRCCs by age group and site.

Colorectal Location	All Patients		Age < 50 years		Age ≥50 years	
**Cause-Specific Survival % (95% CI)**	**Adenocarcinoma**	**Signet Ring Cell**	**Adenocarcinoma**	**Signet Ring Cell**	**Adenocarcinoma**	**Signet Ring Cell**
**All Sites**						
**Localized**						
1-year	95.1 (95.0-95.2)	91.1 (88.7-93.1)	99.0 (98.8-99.2)	91.4 (82.7-95.8)	94.7 (94.6-94.9)	91.1 (88.5-93.2)
2-year	92.5 (92.3-92.6)	86.4 (83.5-88.8)	97.7 (97.3-98.0)	87.7 (78.3-93.2)	92.0 (91.8-92.2)	86.2 (83.0-88.8)
5-year	85.8 (85.5-86.0)	77.0 (73.4-80.2)	92.1 (91.5-92.7)	73.9 (62.8-82.1)	85.2 (84.9-85.4)	77.5 (73.7-80.9)
10-year	79.5 (79.2-79.8)	70.7 (66.5-74.4)	86.6 (85.8-87.4)	70.5 (58.8-79.4)	78.8 (78.5-79.1)	70.6 (66.1-74.7)
Median (months)	-	-	-	-	-	-
**Regional**						
1-year	91.7 (91.5-91.8)	79.5 (77.7-81.2)	97.4 (97.2-97.7)	87.7 (84.2-90.4)	90.9 (90.7-91.0)	77.3 (75.2-79.3)
2-year	85.1 (84.9-85.3)	61.5 (59.3-63.6)	92.5 (92.0-92.9)	66.0 (61.3-70.3)	84.1 (83.8-84.3)	60.4 (57.9-62.7)
5-year	70.4 (70.1-70.7)	43.5 (41.3-45.8)	79.1 (78.4-79.7)	44.1 (39.3-48.9)	69.2 (68.9-69.5)	43.4 (40.9-45.9)
10-year	60.8 (60.5-61.1)	36.2 (33.9-38.5)	69.0 (68.1-69.8)	36.3 (31.5-41.2)	59.6 (59.2-59.9)	36.2 (33.6-38.8)
Median (months)	-	38.2	-	43.6	-	36.9
**Distant**						
1-year	57.9 (57.5-58.2)	46.5 (44.3-48.7)	74.2 (73.3-75.1)	56.2 (51.8-60.3)	55.1 (54.6-55.5)	43.1 (40.5-45.6)
2-year	37.3 (36.9-37.7)	22.6 (20.8-24.5)	50.4 (49.4-51.4)	27.8 (24.0-31.8)	35.1 (34.7-35.5)	20.7 (18.6-22.9)
5-year	14.2 (13.9-14.5)	5.8 (4.8-7.0)	19.7 (18.9-20.5)	8.6 (6.3-11.5)	13.2 (12.9-13.5)	4.8 (3.7-6.1)
10-year	8.5 (8.6-9.1)	3.1 (2.3-4.2)	12.5 (11.8-13.3)	4.0 (2.3-6.5)	8.2 (7.9-8.5)	2.8 (1.9-4.0)
Median (months)	16.1	11.1	24.2	14.1	14.6	9.7
**Right Colon**						
**Localized**						
1-year	95.1 (94.9-95.3)	93.1 (90.0-95.3)	99.1 (98.5-99.4)	94.6 (80.1-98.6)	94.9 (94.6-95.1)	92.9 (89.6-95.2)
2-year	92.6 (92.4-92.9)	90.0 (86.5-92.7)	97.5 (96.7-98.2)	94.6 (80.1-98.6)	92.4 (92.1-92.6)	89.5 (85.7-92.4)
5-year	87.1 (86.8-87.5)	83.1 (78.7-86.7)	93.8 (92.6-94.9)	89.2 (73.7-95.8)	86.7 (86.4-87.1)	82.4 (77.6-86.2)
10-year	82.2 (81.7-82.6)	75.6 (70.0-80.2)	90.4 (88.8-91.8)	85.4 (68.0-93.7)	81.7 (81.2-82.2)	74.2 (68.2-79.3)
Median (months)	-	-	-	-	-	-
**Regional**						
1-year	91.5 (89.3-89.9)	78.4 (75.8-80.7)	96.1 (95.4-96.8)	87.9 (81.8-92.0)	89.1 (88.8-89.4)	76.7 (73.9-79.3)
2-year	84.1 (81.4-82.2)	62.3 (59.3-65.1)	89.4 (88.2-90.4)	69.0 (61.3-75.4)	81.2 (80.8-81.6)	61.2 (57.9-64.2)
5-year	70.4 (67.6-68.6)	46.6 (43.6-49.7)	77.3 (75.7-78.8)	48.5 (40.5-56.0)	67.3 (66.8-67.8)	46.4 (43.0-49.6)
10-year	63.2 (60.5-61.6)	38.9 (35.7-42.1)	71.8 (70.1-73.5)	37.4 (29.4-45.3)	60.1 (59.6-60.7)	39.4 (35.9-42.8)
Median (months)	-	45.6	-	53.0	-	44.8
**Distant**						
1-year	49.7 (49.1-50.4)	49.7 (46.8-52.5)	65.5 (63.4-67.6)	60.3 (53.9-66.1)	48.0 (47.3-48.8)	46.8 (43.5-50.0)
2-year	28.7 (28.0-29.3)	24.8 (22.3-27.4)	37.9 (35.8-40.0)	31.7 (25.9-37.6)	27.7 (27.0-28.3)	22.9 (20.1-25.7)
5-year	11.4 (10.5-11.4)	6.0 (4.7-7.6)	15.1 (13.6-16.8)	9.1 (6.3-13.9)	10.5 (10.1-11.0)	4.9 (3.5-6.7)
10-year	7.6 (7.2-8.0)	2.6 (1.7-4.0)	11.0 (9.6-12.6)	3.0 (1.2-6.3)	7.2 (6.8-7.7)	2.6 (1.5-4.1)
Median (months)	11.9	11.9	17.9	15.6	11.2	11.1
**Transverse Colon**						
**Localized**						
1-year	94.5 (94.0-95.0)	94.5 (83.9-98.2)	99.3 (97.9-99.8)	IN	94.2 (93.6-94.7)	94.2 (83.0-98.1)
2-year	92.3 (91.7-93.0)	90.6 (78.9-96.0)	99.1 (97.6-99.7)	IN	91.9 (91.2-92.5)	90.1 (77.8-95.8)
5-year	87.2 (86.3-88.0)	83.8 (70.0-91.6)	95.3 (92.8-97.0)	IN	86.6 (85.7-87.4)	82.9 (68.5-91.2)
10-year	81.9 (80.8-82.9)	77.7 (61.8-87.6)	90.2 (86.4-92.9)	IN	81.3 (80.1-82.4)	76.7 (603-87.0)
Median (months)	-	-	-	IN	-	-
**Regional**						
1-year	89.2 (88.6-89.9)	78.2 (71.3-83.6)	94.6 (92.7-95.9)	89.7 (71.3-96.5)	88.7 (88.0-89.4)	75.9 (68.1-82.1)
2-year	83.1 (82.2-83.9)	65.1 (57.5-71.7)	89.6 (87.2-91.5)	79.3 (59.6-90.1)	82.4 (81.5-83.2)	62.3 (53.8-69.7)
5-year	70.6 (69.5-71.6)	44.7 (37.0-52.2)	77.6 (74.4-80.4)	48.3 (29.5-64.8)	69.8 (68.7-70.9)	44.2 (35.7-52.4)
10-year	62.8 (61.6-63.9)	38.1 (30.4-45.9)	69.7 (66.1-73.0)	37.1 (20.0-54.3)	62.0 (68.7-70.9)	38.6 (29.9-47.2)
Median (months)	-	42.2	-	50.5	-	40.2
**Distant**						
1-year	51.2 (49.6-52.8)	39.9 (29.7-49.9)	66.6 (62.1-70.6)	53.3 (26.3-74.4)	49.1 (47.4-50.7)	37.3 (26.4-48.2)
2-year	31.7 (30.2-33.2)	20.8 (12.9-29.9)	44.6 (40.1-49.1)	16.7 (2.9-40.2)	29.9 (28.4-31.5)	21.5 (12.9-31.6)
5-year	12.1 (11.0-13.2)	9.4 (4.0-17.6)	22.2 (18.5-26.2)	8.3 (0.5-30.6)	10.6 (9.5-11.8)	10.0 (4.0-19.2)
10-year	8.1 (7.2-9.1)	9.4 (4.0-17.6)	17.1 (13.6-20.9)	8.3 (0.5-30.6)	6.8 (5.8-7.8)	10.0 (4.0-19.2)
Median (months)	12.6	9.2	20.5	12.5	11.6	8.4
**Left Colon**						
**Localized**						
1-year	95.4 (95.2-95.7)	91.7 (83.3-95.9)	99.3 (98.9-99.6)	93.3 (61.3-99.0)	95.0 (94.8-95.3)	91.3 (81.6-96.0)
2-year	93.2 (92.8-93.5)	89.1 (80.2-94.2)	98.3 (97.7-98.8)	86.7 (56.4-96.5)	92.6 (92.3-92.9)	89.7 (79.7-95.0)
5-year	86.9 (86.5-87.3)	78.7 (67.9-86.2)	93.5 (92.4-94.4)	66.0 (36.5-84.3)	86.2 (85.7-86.7)	81.9 (70.3-89.3)
10-year	80.5 (79.9-81.0)	76.4 (64.7-84.6_	87.9 (86.4-89.3)	66.0 (36.5-84.3)	79.2 (79.1-80.2)	79.0 (65.9-87.5)
Median (months)	-	-	-	-	-	-
**Regional**						
1-year	93.0 (92.7-93.2)	82.9 (78.0-86.7)	98.0 (97.5-98.3)	85.9 (76.5-91.7)	92.2 (91.9-92.5)	81.6 (75.6-86.3)
2-year	87.8 (87.4-88.1)	65.2 (59.4-70.4)	94.3 (93.6-95.0)	64.4 (53.2-73.6)	86.7 (86.3-87.1)	65.5 (58.5-71.6)
5-year	73.7 (73.2-74.2)	44.9 (38.9-50.7_	81.1 (79.9-82.2)	43.4 (32.5-53.8)	72.5 (72.0-73.1)	45.6 (38.5-52.5)
10-year	63.2 (62.7-63.8)	38.9 (32.8-44.9)	70.0 (68.5-71.5)	40.2 (29.4-50.8)	62.1 (61.5-62.8)	38.3 (31.1-45.6)
Median (months)	-	42.1	-	45.0	-	38.7
**Distant**						

1-year	63.7 (63.1-64.3)	44.0 (39.1-48.8)	79.0 (77.7-80.3)	51.0 (42.4-58.9)	60.6 (59.9-61.3)	40.4 (34.4-46.2)
2-year	44.8 (44.1-45.4)	22.8 (18.8-27.0)	57.3 (55.6-58.9)	27.6 (20.3-35.3)	42.2 (41.5-42.9)	20.3 (15.7-25.4)
5-year	18.5 (18.0-19.1)	7.4 (4.9-10.5)	23.0 (21.6-24.4)	10.1 (5.5-16.4)	17.6 (17.0-18.2)	6.1 (3.5-9.7)
10-year	11.3 (10.8-11.8)	4.4 (2.3-7.5)	14.4 (13.1-15.8)	6.7 (2.7-13.3)	10.7 (10.1-11.2)	3.4 (1.4-6.9)
Median (months)	20.3	10.4	27.7	12.3	18.5	9.3
**Rectal**						
**Localized**						
1-year	95.0 (94.7-95.2)	83.8 (76.2-89.1)	98.8 (98.4-99.1)	84.6 (64.0-93.9)	94.5 (94.2-94.7)	83.6 (74.9-89.5)
2-year	91.7 (91.3-92.0)	72.5 (63.8-79.4)	97.1 (96.5-97.6)	76.9 (55.7-88.9)	90.9 (90.6-91.3)	71.4 (61.5-79.2)
5-year	82.9 (82.5-83.4)	55.8 (46.6-64.1)	89.8 (88.7-90.8)	53.8 (33.3-70.6)	82.0 (81.5-82.5)	56.4 (46.0-65.6)
10-year	75.1 (74.5-75.7)	50.6 (41.2-59.3)	83.2 (81.8-84.6)	49.0 (28.5-66.6)	74.0 (73.3-74.6)	51.0 (40.3-60.7)
Median (months)	-	-	-	70.8	-	-
**Regional**						
1-year	93.2 (92.9-93.4)	80.5 (76.6-83.7)	98.0 (97.6-98.3)	88.1 (81.7-92.3)	92.2 (91.9-92.5)	77.0 (72.1-81.2)
2-year	86.6 (86.2-86.9)	56.2 (51.6-60.5)	92.9 (92.3-93.6)	60.9 (52.6-68.3)	85.3 (84.9-85.7)	54.0 (48.4-59.3)
5-year	69.8 (69.3-70.3)	35.2 (30.8-39.7)	78.6 (77.5-79.6)	39.1 (31.0-47.1)	68.0 (67.4-68.5)	33.5 (28.3-38.8)
10-year	57.9 (57.3-58.5)	27.6 (23.3-32.1)	66.6 (65.2-67.9)	33.0 (25.1-41.0)	56.1 (55.4-56.7)	25.0 (19.9-30.4_
Median (months)	-	29.7	-	36.3	-	27.8
**Distant**						
1-year	61.9 (61.2-62.6)	40.3 (34.9-45.6)	75.4 (73.9-76.8)	54.0 (44.6-62.5)	58.9 (58.1-59.6)	32.3 (26.0-38.8)
2-year	39.8 (39.8-40.6)	15.3 (11.5-19.5)	51.5 (49.8-53.2)	21.5 (14.5-29.4)	37.2 (36.4-38.0)	11.6 (7.6-16.6)
5-year	13.3 (12.8-13.8)	2.7 (1.2-5.2)	18.6 (17.2-20.0)	5.5 (2.1-11.3)	12.1 (11.5-12.6)	1.3 (0.3-4.0)
10-year	7.5 (7.0-8.0)	2.7 (1.2-5.2)	10.7 (9.5-11.9)	5.5 (2.1-11.3)	6.8 (6.3-7.3)	1.3 (0.3-4.0)
Median (months)	18.1	9.4	24.8	13.5	16.6	7.2

CI, confidence interval; IN, insufficient to calculate. Right colon includes appendix, cecum, ascending colon, and hepatic flexure. Left colon includes splenic flexure, descending colon, and sigmoid. Rectal includes rectosigmoid colon and rectum.

Table S9. Relative survival of colorectal conventional adenocarcinomas and SRCCs by age group and site.

Colorectal Location	All Patients		Age < 50 years		Age ≥50 years	
Relative Survival % (95% CI)	Adenocarcinoma	Signet Ring Cell	Adenocarcinoma	Signet Ring Cell	Adenocarcinoma	Signet Ring Cell
**All Sites**						
**Localized**						
1-year	94.0 (93.8-94.2)	87.0 (83.9-89.6)	98.7 (98.4-99.0)	89.4 (80.4-94.4)	93.6 (93.4-93.8)	86.7 (83.2-89.5)
2-year	92.1 (91.8-92.3)	82.7 (79.0-85.8)	97.2 (96.8-97.6)	86.0 (76.2-91.9)	91.6 (91.4-91.9)	82.2 (78.2-85.6)
5-year	86.4 (86.0-86.7)	74.1 (69.4-78.2)	91.1 (90.3-91.7)	71.8 (60.3-80.5)	86.0 (85.6-86.3)	74.3 (69.1-78.7)
10-year	79.4 (78.9-79.9)	68.7 (62.1-74.4)	85.0 (84.0-85.9)	67.5 (55.1-77.1)	78.9 (78.3-79.4)	68.7 (61.2-75.1)
Median (months)	-	-	-	-	-	-
**Regional**						
1-year	90.7 (90.5-90.9)	77.3 (75.4-79.2)	97.1 (96.8-97.4)	86.8 (83.2-89.6)	89.8 (89.6-90.0)	74.9 (72.6-77.0)
2-year	84.7 (84.4-84.9)	60.0 (57.7-62.1)	91.9 (91.5-92.4)	64.7 (60.0-69.0)	83.6 (83.4-83.9)	58.7 (56.2-61.2)
5-year	70.9 (70.6-71.3)	42.6 (40.1-44.9)	78.1 (77.4-78.8)	41.9 (37.1-46.6)	69.9 (69.6-70.3)	42.8 (40.0-45.6)
10-year	61.1 (60.7-61.5)	34.9 (32.1-37.7)	67.6 (66.8-68.5)	33.5 (28.7-38.3)	60.2 (59.7-60.7)	35.3 (32.0-38.7)
Median (months)	-	36.6	-	41.1	-	36.1
**Distant**						
1-year	56.8 (56.5-57.2)	45.4 (43.2-47.5)	73.5 (72.6-74.4)	56.0 (51.6-60.1)	54.0 (53.6-54.4)	41.6 (39.1-44.1)
2-year	36.6 (36.2-37.0)	21.6 (19.8-23.5)	49.8 (48.8-50.8)	27.1 (23.3-30.9)	34.4 (34.0-34.8)	19.7 (17.7-21.8)
5-year	13.8 (13.5-14.1)	5.2 (4.3-6.4)	19.1 (18.3-19.9)	7.8 (5.6-10.4)	12.9 (12.6-13.2)	4.3 (3.3-5.5)
10-year	8.6 (8.3-8.8)	3.0 (2.1-4.1)	11.9 (11.1-12.6)	3.6 (2.0-5.8)	8.0 (7.7-8.3)	2.8 (1.8-4.1)
Median (months)	15.6	10.5	23.9	14.0	14.0	9.2
**Right Colon**						
**Localized**						
1-year	94.1 (93.8-94.4)	90.6 (86.4-93.5)	98.8 (98.1-99.3)	94.7 (79.9-98.7)	93.9 (93.5-94.2)	90.1 (85.6-93.3)
2-year	92.8 (92.4-93.2)	86.8 (82.0-90.4)	97.3 (96.4-98.0)	94.7 (79.9-98.7)	92.6 (92.2-93.0)	85.9 (80.6-89.9)
5-year	88.9 (88.3-89.5)	80.1 (73.5-85.3)	93.1 (91.7-94.3)	87.5 (70.2-95.1)	88.7 (88.0-89.3)	79.1 (71.9-84.7)
10-year	83.4 (82.4-84.4)	73.4 (63.9-80.8)	88.4 (86.3-90.1)	80.9 (60.5-91.4)	83.1 (82.1-84.2)	72.4 (61.7-80.6)
Median (months)	-	-	-	-	-	-
**Regional**						
1-year	88.7 (88.4-89.1)	75.8 (73.1-78.3)	95.7 (94.9-96.4)	87.7 (81.6-91.9)	88.2 (87.8-88.5)	73.8 (70.7-76.6)
2-year	81.8 (81.3-82.2)	61.1 (58.0-64.0)	88.6 (87.4-89.7)	69.3 (61.6-75.7)	81.2 (80.7-81.7)	59.7 (56.3-62.9)
5-year	69.8 (69.2-70.3)	46.0 (42.5-49.3)	76.1 (74.5-77.7)	45.4 (37.5-52.9)	69.2 (68.6-69.9)	46.2 (42.3-49.9)
10-year	62.6 (61.8-63.4)	38.5 (34.3-42.7)	69.8 (67.9-71.6)	33.3 (25.6-41.1)	62.0 (61.1-62.9)	39.9 (35.0-44.7)
Median (months)	-	44.3	-	48.1	-	44.3
**Distant**						
1-year	48.6 (47.9-49.3)	48.4 (45.5-51.2)	64.7 (62.6-66.8)	59.9 (53.5-65.6)	46.9 (46.2-47.6)	45.2 (42.0-48.5)
2-year	28.0 (27.4-28.6)	24.0 (21.5-26.5)	37.3 (35.2-39.4)	31.0 (25.4-36.8)	27.0 (26.4-27.6)	22.0 (19.4-24.8)
5-year	10.7 (10.3-11.2)	5.6 (4.3-7.1)	14.6 (13.1-16.2)	8.9 (5.8-12.9)	10.3 (9.9-10.8)	4.6 (3.3-6.3)
10-year	7.5 (7.0-7.9)	2.6 (1.6-4.0)	10.3 (8.9-11.9)	2.6 (1.0-5.6)	7.2 (6.7-7.7)	2.7 (1.5-4.4)
Median (months)	11.5	11.5	17.6	15.2	10.7	10.1
**Transverse Colon**						
**Localized**						
1-year	93.0 (92.2-93.7)	90.5 (78.1-96.1)	99.3 (97.5-99.8)	IN	92.5 (91.7-93.3)	90.0 (76.9-95.8)
2-year	91.8 (90.8-92.6)	88.0 (71.7-95.2)	99.1 (97.0-99.7)	IN	91.3 (90.2-92.2)	87.3 (70.2-94.9)
5-year	86.7 (85.3-88.0)	84.6 (60.1-94.7)	94.6 (91.5-96.6)	IN	86.2 (84.6-87.5)	83.8 (58.5-94.3)
10-year	79.8 (77.5-81.8)	64.6 (34.5-83.6)	89.5 (85.1-92.7)	IN	79.1 (76.6-81.3)	62.8 (32.2-82.6)
Median (months)	-	-	-	IN	-	-
**Regional**						
1-year	87.0 (86.1-87.7)	77.5 (70.2-83.2)	93.8 (91.8-95.3)	90.2 (72.1-96.8)	86.3 (85.4-87.1)	75.0 (66.6-81.5)
2-year	81.5 (80.5-82.4)	64.1 (56.0-71.1)	88.5 (86.0-90.6)	80.3 (60.9-90.7)	80.8 (79.7-81.8)	60.8 (51.7-68.8)
5-year	69.8 (68.5-71.0)	42.3 (34.0-50.5)	75.5 (72.3-78.5)	47.1 (28.6-63.5)	69.2 (67.8-70.5)	41.5 (32.1-50.7)
10-year	60.4 (58.7-62.1)	33.0 (24.8-41.4)	67.5 (63.7-71.0)	36.3 (19.5-53.4)	59.6 (57.7-61.5)	32.2 (23.0-41.8)
Median (months)	-	38.2	-	50.1	-	35.5
**Distant**						
1-year	50.4 (48.8-52.0)	38.1 (28.3-47.8)	66.1 (61.7-70.2)	56.4 (29.6-76.4)	48.2 (46.5-49.9)	34.4 (24.1-45.0)
2-year	31.1 (29.6-32.6)	18.4 (11.2-26.9)	43.9 (39.4-48.3)	12.5 (2.1-32.9)	29.3 (27.7-30.9)	19.6 (11.6-29.2)
5-year	11.8 (10.7-13.0)	6.6 (2.5-13.3)	21.3 (17.6-25.1)	6.3 (0.4-24.8)	10.4 (9.4-11.6)	6.7 (2.3-14.5)
10-year	8.0 (6.9-9.1)	6.6 (2.5-13.3)	15.9 (12.5-19.6)	6.3 (0.4-24.8)	6.8 (5.7-7.9)	6.7 (2.3-14.5)
Median (months)	12.2	8.9	20.0	13.0	11.1	7.2
**Left Colon**						
**Localized**						
1-year	94.0 (93.6-94.3)	82.3 (72.1-89.0)	98.7 (98.2-99.1)	93.4 (60.8-99.1)	93.5 (93.1-93.9)	80.0 (68.4-87.8)
2-year	92.2 (91.8-92.6)	80.4 (69.6-87.7)	97.7 (96.9-98.2)	87.1 (55.9-96.8)	91.6 (91.2-92.1)	78.3 (66.2-86.5)
5-year	86.9 (86.2-87.5)	72.1 (59.6-81.4)	92.2 (91.0-93.3)	67.0 (36.6-85.3)	86.3 (85.6-87.0)	71.8 (57.4-82.0)
10-year	79.8 (78.8-80.7)	68.6 (47.7-82.7)	86.1 (84.3-87.7)	67.0 (36.6-85.3)	79.1 (78.1-80.1)	68.9 (43.1-84.8)
Median (months)	-	-	-	-	-	-
**Regional**						
1-year	91.8 (91.5-92.1)	80.4 (75.2-84.6)	97.7 (97.2-98.1)	84.5 (75.1-90.6)	90.9 (90.5-91.3)	78.7 (72.2-83.8)
2-year	87.1 (86.7-87.5)	61.9 (55.9-67.4)	93.8 (93.0-94.5)	61.0 (50.0-70.3)	86.1 (85.6-86.5)	62.4 (55.0-68.8)
5-year	73.7 (73.2-74.3)	43.5 (37.4-49.6)	80.1 (78.8-81.2)	40.2 (29.8-50.4)	72.7 (72.1-73.4)	45.1 (37.4-52.5)
10-year	63.6 (62.8-64.3)	35.6 (28.8-42.4)	68.8 (67.3-70.3)	36.2 (25.8-46.7)	62.7 (61.9-63.6)	35.2 (26.6-43.9)
Median (months)	-	37.9	-	39.0	-	36.1
**Distant**						

1-year	62.7 (62.0-63.3)	43.1 (38.3-47.9)	78.4 (77.1-79.7)	50.4 (41.9-58.4)	59.5 (58.7-60.2)	39.4 (33.5-45.2)
2-year	44.1 (43.4-44.8)	21.6 (17.7-25.7)	56.8 (55.1-58.4)	27.0 (19.8-34.6)	41.5 (40.8-42.3)	18.8 (14.4-23.8)
5-year	18.1 (17.6-18.7)	6.1 (3.9-9.0)	22.3 (20.9-23.7)	8.4 (4.3-14.2)	17.3 (16.7-17.9)	5.0 (2.7-8.4)
10-year	10.9 (10.3-11.4)	3.7 (1.9-6.7)	13.7 (12.4-15.1)	5.6 (2.2-11.5)	10.3 (9.7-10.9)	2.9 (1.1-6.4)
Median (months)	19.8	10.0	27.5	12.1	18.0	8.8
**Rectal**						
**Localized**						
1-year	94.1 (93.8-94.4)	78.6 (70.2-84.8)	98.6 (98.0-98.9)	78.8 (58.5-90.0)	93.5 (93.2-93.9)	78.5 (68.8-85.5)
2-year	91.2 (90.7-91.6)	69.6 (60.4-77.0)	96.6 (95.8-97.2)	71.8 (51.1-84.9)	90.5 (90.0-90.9)	68.9 (58.3-77.4)
5-year	83.0 (82.4-83.6)	52.8 (43.1-61.6)	88.7 (87.5-89.8)	50.5 (30.9-67.2)	82.3 (81.6-82.9)	53.4 (42.1-63.4)
10-year	74.6 (73.7-75.5)	51.1 (41.0-60.3)	81.7 (80.1-83.2)	46.3 (26.7-63.7)	73.6 (72.6-74.5)	52.4 (39.5-63.8)
Median (months)	-	-	-	70.2	-	-
**Regional**						
1-year	92.5 (92.2-92.8)	78.9 (74.9-82.4)	97.8 (97.3-98.1)	86.5 (79.9-91.0)	91.5 (91.1-91.8)	75.5 (70.4-80.0)
2-year	86.1 (85.7-86.5)	54.8 (50.1-59.2)	92.6 (91.9-93.2)	58.9 (50.6-66.3)	84.8 (84.4-85.3)	52.9 (47.2-58.3)
5-year	69.9 (69.4-70.5)	34.2 (29.8-38.8)	77.9 (76.8-79.0)	38.1 (30.2-46.0)	68.3 (67.7-68.9)	32.5 (27.1-37.9)
10-year	57.7 (56.9-58.4)	26.2 (21.6-31.0)	65.5 (64.1-66.9)	31.6 (23.8-39.7)	56.0 (55.2-56.8)	23.5 (18.0-29.5)
Median (months)	-	28.0	-	34.4	-	27.3
**Distant**						
1-year	61.0 (60.3-61.7)	39.6 (34.3-44.8)	74.6 (73.1-76.0)	54.2 (44.9-62.6)	57.9 (57.1-58.7)	31.2 (25.1-37.6)
2-year	39.1 (38.4-39.8)	14.3 (10.8-18.4)	50.8 (49.1-52.5)	20.9 (14.1-28.7)	36.4 (35.6-37.2)	10.6 (6.9-15.2)
5-year	12.8 (12.3-13.3)	2.6 (1.1-5.0)	18.1 (16.8-19.5)	5.4 (2.1-11.1)	11.6 (11.0-12.1)	1.2 (0.2-3.7)
10-year	7.3 (6.8-7.7)	2.6 (1.1-5.0)	10.1 (9.0-11.4)	5.4 (2.1-11.1)	6.6 (6.1-7.1)	1.2 (0.2-3.7)
Median (months)	17.7	9.1	24.4	13.5	16.1	6.7

CI, confidence interval; IN, insufficient to calculate. Right colon includes appendix, cecum, ascending colon, and hepatic flexure. Left colon includes splenic flexure, descending colon, and sigmoid. Rectal includes rectosigmoid colon and rectum.
